# Community exposure and vulnerability to water quality and availability: a case study in the mining-affected Pazña Municipality, Lake Poopó Basin, Bolivian Altiplano

**DOI:** 10.1007/s00267-017-0893-5

**Published:** 2017-06-08

**Authors:** Megan French, Natalie Alem, Stephen J. Edwards, Efraín Blanco Coariti, Helga Cauthin, Karen A. Hudson-Edwards, Karen Luyckx, Jorge Quintanilla, Oscar Sánchez Miranda

**Affiliations:** 1 Institute for Risk and Disaster Reduction, University College London, Gower Street, London, WC1E 6BT UK; 2 Centro de Comunicación y Desarrollo Andino, Avenida Tadeo Haenke No. 2231, Cochabamba, Bolivia; 3 UCL Hazard Centre, Department of Earth Sciences, University College London, Gower Street, London, WC1E 6BT UK; 4 Andean Risk & Resilience Institute for Sustainability & the Environment, Cerro El Plomo, Las Condes, 5630 Santiago Chile; 5 Instituto de Investigaciones Químicas, Universidad Mayor San Andrés, 303 La Paz, Bolivia; 6 Department of Earth and Planetary Sciences, Birkbeck, University of London, Malet Street, London, WC1E 7HX UK; 7 Catholic Agency for Overseas Development, 55 Westminster Bridge Road, London, SE1 7JB UK; 8 Feedback, 61 Mare Street, London, E8 4RG UK

**Keywords:** Water resources management, Water quality, Water scarcity, Vulnerability, Bolivian Altiplano, Mining

## Abstract

Assessing water sources for drinking and irrigation along with community vulnerability, especially in developing and rural regions, is important for reducing risk posed by poor water quality and limited water availability and accessibility. We present a case study of rural mining-agricultural communities in the Lake Poopó Basin, one of the poorest regions on the Bolivian Altiplano. Here, relatively low rainfall, high evaporation, salinization and unregulated mining activity have contributed to environmental degradation and water issues, which is a situation facing many Altiplano communities. Social data from 72 households and chemical water quality data from 27 surface water and groundwater sites obtained between August 2013 and July 2014 were used to develop locally relevant vulnerability assessment methodologies and ratings with respect to water availability and quality, and Chemical Water Quality Hazard Ratings to assess water quality status. Levels of natural and mining-related contamination in many waters (CWQHR ≥ 6; 78% of assessed sites) mean that effective remediation would be challenging and require substantial investment. Although waters of fair to good chemical quality (CWQHR ≤ 5; 22% of assessed sites) do exist, treatment may still be required depending on use, and access issues remain problematic. There is a need to comply with water quality legislation, improve and maintain basic water supply and storage infrastructure, build and operate water and wastewater treatment plants, and adequately and safely contain and treat mine waste. This study serves as a framework that could be used elsewhere for assessing and mitigating water contamination and availability affecting vulnerable populations.

## Introduction

Assessment of water sources used for potable water, irrigation and livestock along with assessment of community vulnerability are key steps in reducing risk posed by poor water quality and inadequate availability. In vulnerable situations where communities lack the ability to anticipate, resist, survive and recover from any impact of a hazard (Blaikie et al. 1994), there can be a resultant risk—defined here as the product of hazard and vulnerability—to the population and the ecosystems and environments upon which they depend. This study uses the term hazard to refer to stresses arising from chemical water quality and water availability. Water quality is central to human, animal and ecosystem health; many chemical (e.g., salts, nutrients and metals) and biological (e.g., micro-organisms) constituents can pose potential hazards when at elevated concentrations according to exposure and dose (i.e., ingestion of water and food, inhalation, adsorption to skin; WHO 2011). A potential hazard also exists in situations of inadequate water availability (due to limited resource and/or lack of supply infrastructure) to meet basic requirements (e.g., for consumption and sanitation) and/or the needs of activities such as food production (i.e., irrigation).

Assessment and monitoring of water resources and their quality is generally routine management protocol in developed countries. However, this is not always the case in developing regions or remote areas where vulnerable communities are disproportionately affected by poor water quality (UNEP 2010). Areas suffering from limited water resource availability (e.g., arid regions; Mohsen 2007) or accessibility (e.g., lack of boreholes and pipelines; Bonsor et al. 2011) can also be particularly vulnerable to water risk. This is especially the case if limited water resources are compromised by natural and anthropogenic contamination (e.g., brackish groundwater, high metal concentrations, wastewater and industrial effluent) or changing climate (e.g., glacial recession; Vergara et al. 2007). Additional susceptibilities can include poverty (e.g., Hanjra et al. 2009), high sector water usage (e.g., irrigation; Cai et al. 2003), remoteness (e.g., Garrett et al. 2008), insufficient water management and lack of treatment facilities or supply infrastructure, lack of education and significant presence of sensitive groups (e.g., children and the elderly). Communities affected by such factors may benefit from support in assessment of their degree of vulnerability and water situation, both in terms of climate change adaptation and with regards to health and sanitation, particularly given that improving access to safe water can be an effective part of poverty alleviation strategies (WHO 2011).

**Fig. 1:**
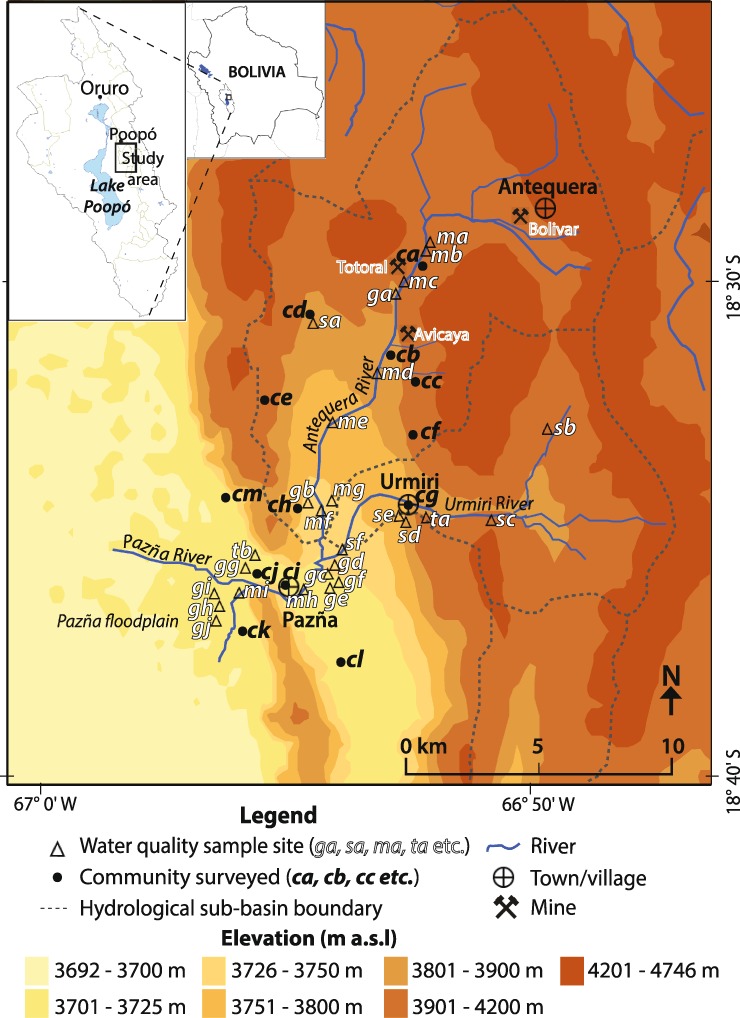
Elevation map (m a.s.l) showing study area, communities surveyed (*circles*; *ca-cm*) and water quality sample sites (*triangles* and codes; *ga-gj* groundwater well; *sa-sf* mining-separated surface source; *ma-mi* mining-associated surface source; *ta-tb* thermal water). Inset map shows location of study area within the Lake Poopó Basin and within Bolivia (500 m resolution, SRTM data, 2000)

Components of water risk are prevalent in the Lake Poopó Basin (~3685 m above sea level (a.s.l.), 24,013 km^2^, 66° 18 ‘ −67° 56’ W; 17° 07 ‘ −20° 01’ S, Fig. 1 inset), one of the poorest regions on the Bolivian Altiplano, where many communities lack access to safe drinking water and sanitation. This largely rural region is characterised by an historical and on-going legacy of unregulated mineral mining. This combined with a poor socio-economic situation, relatively low rainfall and high evaporation, have contributed to environmental degradation and water issues (e.g., Calizaya 2009). The Lake Poopó Basin is confronted with water scarcity in the dry season (as evidenced by the lake drying out in December 2015, National Geographic 2016), periodic and heavy rainfall during the wet season, considerable water quality issues due to anthropogenic (e.g., mining) and natural sources (mineral reactions and thermal springs), as well as climatic factors (high evaporation and salinization) (Abarca-Del Rio et al. 2012; Calizaya 2009; CAMINAR 2013; Ekdahl 2007; García et al. 2010; Klartell and Sandholm 2009; Lilja and Linde 2006; Navarro Torres et al. 2012; Pillco and Bengtsson 2006; Quintanilla et al. 2012; Ramos Ramos et al. 2012, 2014; Rosenberg and Stålhammer 2010; Selander and Svan 2007; Tapia et al. 2012; Tapia and Audry 2013; Van Den Bergh et al. 2010).

In this paper we report on the evaluation of the exposure and vulnerability to the hazards of water availability and chemical quality in communities of Pazña Municipality (sub-basins of Antequera and Urmiri and the adjacent Pazña floodplain of the Lake Poopó Basin; Fig. 1). The key to this study is the unique collaboration between UK and Bolivian universities and non-government organisations (NGOs) to examine a complex problem through the integration of natural and social sciences. Through this collaboration and engagement with communities it has been possible to evaluate water risk in the area by developing and applying some simple methods to measure hazard and vulnerability in ways that are relatively easy for end-users to understand. This has led to the development of a number of evidence-based recommendations from community to government level and acts as a case study that could be transposed to similar environments and situations elsewhere to aid evidence-based water risk assessments.

## Background

### Environmental Setting

The study area is located in Pazña Municipality on the central eastern margin of the Lake Poopó Basin, ~60 km south east of the city of Oruro (Fig. 1 inset), and includes two sub-basins (Antequera, 150.4 km^2^ and Urmiri, 76.2 km^2^; Quintanilla et al. 2012) (Fig. 1). The area is a rural region located at elevations of ~3700–4750 m a.s.l. and is semi-arid with relatively high evaporation (e.g., 323 mm/y in Antequera sub-basin; Calizaya 2009) and relatively low seasonal rainfall. The local rainfall record in Poopó Village in the adjacent sub-basin to the study area (Fig. 1) in 2013 was 470.8 mm/y, and nearly 70% of this occurred in December, January and February (SENAMHI 2014; Fig. S1). Minimum average temperatures range from around −3 °C in winter to ~7 °C in summer, and maximum average temperatures are ~15 °C in winter to ~20 °C in summer (Fig. S1). These factors result in limited surface water availability in the dry season (Calizaya 2009; Pillco and Bengtsson 2006), salinization of soils and seasonal frost, which, along with erosion, soil compaction, loss of vegetation cover and loss of soil fertility (Calizaya 2009; Quintanilla et al. 2012; Orsag 2009), limit agricultural and ranching activities, the main economic activities in rural Altiplano communities.

The regional geology comprises steeply folded Palaeozoic rocks, which form parallel bands with north-westerly strike directions (Sergeotecmin 1992). Overlying this are Quaternary deposits that include fluvial sediments, aeolian sediments, and lacustrine deposits that correspond to the paleolakes that once covered the Altiplano (Argollo and Mourguiart 2000; Wirrmann and Mourguiart 1995). The main hydrogeological unit of the wider Lake Poopó Basin is a Quaternary aquifer composed of sediments that overlie low permeability Palaeozoic sedimentary rocks (e.g., shale and sandstone) (Quintanilla et al. 2012). The recharge zone is located in the hills of the Eastern cordillera and at mouths of the valleys, although the rate of groundwater recharge is understood to be generally very low (Montes de Oca 1997). The approximate regional groundwater flow is north to south. Generalised hydrogeological information for the study area from SERGEOMIN (2000) indicates that the aquifers in the upstream regions of the Antequera and Urmiri sub-basins are thought to be insignificant, and in downstream areas before and after the confluence with the Pazña River a large productive aquifer is indicated. The downstream area of the Pazña River and the majority of the remaining municipality of Pazña are together indicated as a small aquifer of limited production. The study area encompasses two contrasting rivers (Fig. 1): the Urmiri River without mining activity, but with inputs from natural thermal waters; the Antequera River that has several mines along its upper reach, and is affected by acid mine drainage (AMD) and significant informal (historical and new) tailing heaps within the channel (CAMINAR 2013).

Mining contributes 31.1% of the GDP of the Department of Oruro (INE 2013) in comparison to 6.66% of Bolivian GDP in 2013 (INE 2014). There is a history of unregulated mining activity that has affected the environment both aesthetically (tailing heaps) and due to contamination of soil and water (Quintanilla et al. 2012). Poly-metallic and metalloid deposits may contain Cu, Sn, Au, Ag, Sb, W, Bi, Zn and Pb. The most abundant non-metallic mineral is sodium chloride salt (NaCl), which is often associated with high concentrations of Li, B, K and Mg. Other common salt deposits include gypsum, potassium carbonate, sodium carbonate and sodium sulphate (UNEP 1996).

### Socio-Economic Context and Local Water Usage

In 2012, 59.7% of the population of the Municipality of Pazña (5853 inhabitants) was considered as poor (48.3% moderate, 10.8% indigent, 0.6% marginal poverty) (Census 2012). The main occupations at this time were in agriculture, livestock^1^, forestry and fishery (collectively 46.1% of the labour force) (Census 2012). Other occupational groups were services (11.0%) and construction, manufacturing and industry (10.8%). The municipality and surrounding area has been a centre for mining since at least the 16th Century, with a focus on Ag up to the 1890s and afterwards Sn. The political and economic strength of the mining sector at a national level results in an unequal power distribution in relation to the local communities (LIDEMA 2008).

In the wider Lake Poopó Basin the main uses of water are for agricultural irrigation (8.1 m s^−1^), domestic/household use and consumption (0.2 m s^−1^), and mining, industry and livestock watering (0.5–0.6 m s^−1^) (Calizaya 2009). There are no water meters in the rural areas, and in remote communities there are no official records of usage or abstraction. In Pazña Municipality, 55.3% of households were recorded as receiving water from pipe networks (Census 2012). The (informal) piped network transfers chlorinated surface water (spring water, or wetland water in the case of Pazña Village) (Zacarías Ortega, oficial mayor técnico del Municipio de Pazña *pers. comm*. February 2015) to communities for drinking and domestic purposes. Some communities are served by the municipality, whilst others have an independent piped network built either by the Government’s Proyecto Mi Agua or the NGO Visión Mundial. Communities not receiving piped water largely obtain (supposedly untreated) water from groundwater wells and/or directly from springs and/or rivers (Quintanilla et al. 2012). Most irrigation is dominantly rain-fed during the wet season, and livestock watering is generally from well water or rivers.

Census (2012) reports that only 41.8% of households within the entire Pazña Municipality receive any kind of sewage service, but for our study area, García (2011) reports that there are no operational wastewater treatment systems other than some septic tanks. Although wastewater lagoons were developed in Pazña Village, the system is not in operation, and it would seem that there are no operational septic tanks other than in some mining centres. Consequently, untreated wastewater is discharged, for example, to the upper Antequera River (García 2011). The mining communities that do have systems in operation include Bolívar, where septic tanks and anaerobic filters are supposedly used, but after separation of solids the residue water is believed to be discharged untreated to the Antequera River (Ekdahl 2007).

## Methods and Materials

### Assessment of Community Vulnerability

Vulnerability is understood here as the ability of a person or group of persons to anticipate, resist, survive and recover from the impact of a hazard (Blaikie et al. 1994), which we consider here to be water quality and availability. In developing our vulnerability methodology we draw on various approaches to incorporate elements of the physical resistance of the population (i.e., damage to life and health), economic activity and damages, and political, social, cultural, and ecological aspects. We also draw on numerous studies (Ávila 2004; Barrenechea et al. 2000; Cardona 2003; Cutter 1996; Cutter et al. 2003; Eakin and Luers 2006; INDECI 2006; Madrigal 2011; Perles et al. 2009; Perona and Rocchi 2001; SDC and PROMIC 2006; Wisner and Luce 1993) in order to select and define our vulnerability indicators (vi; Table S1), which, as with other studies, are largely defined according to the study’s objectives and the definition of vulnerability used.

Fieldwork was carried out in the study area during October and December 2013, and involved 72 household surveys (250 residents; 55.6% female and 44.4% male) within 13 communities (coded here as *ca–cm*, Fig. 1, Table 1) and interviews with 10 key informants (government officials, community and institutional leaders, local authorities, staff of health centres, and teachers and principals of educational units) within Pazña Municipality. Involvement in the survey was largely dependent upon willingness and availability, but effort was made to solicit response in towns and remote communities. We appreciate that this factor limits somewhat the survey data as we have not necessarily obtained a representative sample of the population, rather those available and/or interested. The surveyed population of 250 represents 6.8% of the total population of communities studied (3681 inhabitants) and 4.3% of the total population of the municipality (5853 inhabitants; Census 2012).

**Table 1:** Vulnerability component (Vc) and total vulnerability (Vt) scores for each community and for the study area (based on averaged scores for households within each community or the study area; Table S2 for household scores and Fig. 1 for community locations)

Community code and name (surveyed household code)														
	*ca*	*cb*	*cc*	*cd*	*ce*	*cf*	*cg*	*ch*	*ci*	*cj*	*ck*	*cl*	*Cm*	*ca - cm*
	Totoral (h1−h12)	Avicaya (h13−h20)	Santa Rosa (h21−h24)	Kuchu Avicaya (h25)	San Ignacio (h26−h31)	Sakani (h32)	Urmiri (h33−h36)	Vilaque (h37−h39)	Pazña (h40−h62)	San Martín (h63−h64)	Santa Filomena (h65−h70)	Canaslupe (h71)	Ocuri Chico (h72)	Study area (h1−h72)
Total population^a^	1128	616	27	41	68	15	168	7	1407	11	36	142	15	3681
Number of people surveyed	53	33	11	2	16	3	10	7	72	6	31	4	2	250
Number households surveyed	12	8	4	1	6	1	4	3	23	2	6	1	1	72
Average no. people/household	4.4	4.1	2.8	2.0	2.7	3.0	2.5	2.3	3.1	3.0	5.2	4.0	2.0	3.5
Vc1(vi1–vi5): Environmental^b^	6.41	5.69	5.44	7.01	6.88	8.90	5.70	7.58	5.93	6.89	7.42	5.18	7.32	6.31
Vc1 weighted to Vt	1.72	1.52	1.46	1.88	1.84	2.39	1.53	2.03	1.59	1.85	1.99	1.39	1.96	1.69
Vc2 (vi6 –vi14): Exposure	6.96	7.11	5.27	3.57	4.17	4.82	4.96	5.26	5.28	4.23	6.15	4.46	4.46	5.64
Vc2 weighted to Vt	1.91	1.95	1.45	0.98	1.14	1.32	1.36	1.44	1.45	1.16	1.69	1.23	1.23	1.55
Vc3 (vi15–vi18): Political & institutional	7.54	7.66	8.44	7.16	8.11	8.30	7.59	7.73	8.52	8.86	7.82	7.73	8.30	8.05
Vc3 weighted to Vt	1.08	1.10	1.21	1.03	1.17	1.19	1.09	1.11	1.22	1.27	1.12	1.11	1.19	1.16
Vc4 (vi19–vi21): Educational & cultural	4.87	6.01	4.79	2.50	5.76	10.00	3.85	5.56	6.07	4.58	4.63	6.53	4.58	5.45
Vc4 weighted to Vt	0.57	0.71	0.56	0.29	0.68	1.18	0.45	0.65	0.71	0.54	0.54	0.77	0.54	0.64
Vc5 (vi22–vi24): Social	4.22	5.29	4.02	2.50	3.65	2.81	4.41	4.84	5.20	5.23	4.30	4.38	2.50	4.60
Vc5 weighted to Vt	0.44	0.55	0.42	0.26	0.38	0.29	0.46	0.51	0.54	0.55	0.45	0.46	0.26	0.48
Vc6 (vi25–vi28): Economic	6.52	6.61	6.88	6.07	7.83	6.07	6.88	6.37	7.35	5.54	6.67	6.96	6.96	6.92
Vc6 weighted to Vt	0.60	0.60	0.63	0.56	0.72	0.56	0.63	0.58	0.67	0.51	0.61	0.64	0.64	0.63
Vt: Total vulnerability^c^	6.32	6.44	5.73	5.00	5.93	6.93	5.52	6.33	6.20	5.87	6.41	5.59	5.82	6.15

^a^ Census (2012) data

^b^ Example for *ca*: Vc1 *ca* *=* (mean vi h1−h12/4 × vi1 wf/Vc1 wf] + …[mean vi5 h1−h12/4 × vi5 wf/Vc1 wf]) × 10

Where wf: weighting factor on scale 1 (least)−10 (highest) of relative importance of vis to each other (refer to Table S1)

^c^ Example for *ca*: Vt *ca* *=* ([mean vi1 h1−h12/4 × vi1 wf/Vt wf] + …[mean vi28 h1−h12/4 × vi28 wf/Vt wf]) × 10

**Fig. 2:**
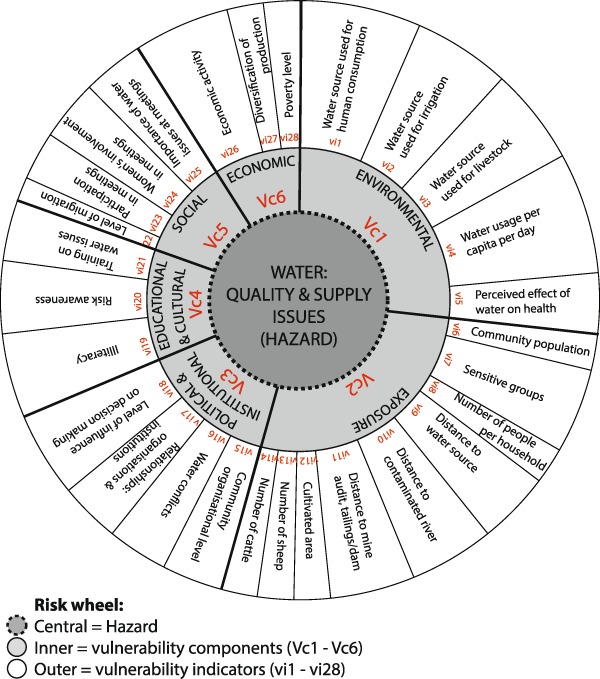
Risk wheel illustrating the vulnerability components (Vc) and vulnerability indicators (vi), and their weightings, used to determine total vulnerability (Vt) to water scarcity and contamination

In order to estimate the degree of vulnerability to water quality and availability, household survey results were used in conjunction with Census data on municipality population figures, poverty rating and provision of piped water network and sewage services (Census 2012). Together these provided responses to 28 vi (Fig. 2), whereby households scored 1, 2, 3 or 4 (i.e., rating low, medium, high or very high; Table S1) for each vi depending on their responses (e.g., vi = 1 for a household next to their water source, versus vi = 4 if the water is > 1000 m from the house) or their community situation. Household vi results (Table S2) were then used to calculate six vulnerability components (Vc) and total vulnerability (Vt) for each household, and subsequently each community and in turn the entire study area. The Vcs and respective vis are as follows: Vc1 environmental (vi1–vi5), Vc2 exposure (vi6–vi14), Vc3 political and institutional (vi15–vi18), Vc4 educational and cultural (vi19–vi21), Vc5 social (vi22–vi25), and Vc6 economic (vi26–vi28) (see Supplementary Information). The proportional representation of each vi to their respective Vcs and to the Vt is illustrated by Fig. 2.

In order to calculate Vcs and Vts, each vi was assigned a weighing factor (wf) of between 1 (least) and 10 (highest) to represent the relative importance of the indicators to each other (Fig. 2; Table S1), whereby wfs were decided upon using information from interviews with key informants and by general knowledge gained during the research. The determination of each household (community and study area) Vc (and Vt) was then undertaken by dividing each household vi score (or mean community vi, or mean study area vi) by the maximum possible score of four and multiplying this by a ratio of the respective vi wf to the sum of the wf of each Vc, i.e., Vc wf (or Vt wf). The calculated values for each vi associated with each Vc (or all 28 vis for Vt) were then summed before multiplying by 10 to fall into a 0–10 vulnerability rating (i.e., scale of zero to very high for consistency with the Chemical Water Quality Hazard Ratings (CWQHR scale), as shown in Eq. 1 using Vc1 for h1 as an example, and Eq. 2 to illustrate the calculation of Vt for the entire study area (i.e., *ca−cm* = h1−h72).

1 $$Vc1\;h1 =	\ ( [ vi1\;h1/4 \times vi1\;wf/Vc1\;wf ]\\ 	+ \ldots [ vi5\;h1/4 \times vi5\;wf/Vc1\;wf ] )\, \times \,10$$

2 $$Vt \;ca - cm = \ ([ {mean\;vi1\;h1 - h72/4 \times vi1\;wf/Vt\;wf} ]\\ \ + \ldots [ {mean\;vi28\;h1 - h72/4 \times vi28\;wf/Vt\;wf} ]) \times 10$$

Subsequently, Vc1–Vc6 and Vt values were obtained for each of the 72 households, the communities and the entire study area (Table 1 and Table S2). The 0–10 rating of resultant Vt scores expresses overall vulnerability to water quality and scarcity issues and can be interpreted in a general way using the following vulnerability levels; low (<2.5), medium (2.5–4.9), high (5.0–7.4) and very high (>7.5) (after INDECI 2006; Table S3). The details and definitions of the Vcs contributing to Vt are summarised in the Supplementary Information.

### Water Sampling and River Flow Measurements

Water quality sampling was undertaken in four periods to reflect seasonal variation in climate: (i) August 13–16th 2013, (ii) December 16–20th 2013, (iii) April 7–12th 2014 and (iv) July 9–13th 2014. A total of 27 surface water and groundwater sites were sampled to assess chemical quality and provenance (Fig. 1). Sampling was performed by first collecting water in a bucket (that had first been rinsed three times with sample water) and allowing the water to stand for a few minutes to allow suspended material to settle out. In-situ measurements were taken using a Multi-parameter HI 9828 (Hanna Instruments) from the bucket water for pH, temperature, alkalinity, electrical conductivity (EC, measured as specific conductivity automatically adjusted for 25 °C) and total dissolved solids. A 50 mL syringe pre-washed with Milli-Q water was used to take water from the bucket and this was filtered through a 0.45 µm filter cartridge into four clean 30 mL plastic vials; two for cations and two for anions (one set for analysis at UCL and the other for analysis at Instituto de Investigaciones Químicas, UMSA). A few drops of 50% nitric acid were added to two vials containing water to be analysed for cations. Field blank samples were also prepared using Milli-Q water. Vials were labelled and sealed, then stored in a cooler. One set of samples were taken to UMSA and the other set were shipped to the UK for analysis. We report here the results of the UK analysis.

Assessment of water quantity involved measuring the water levels using a standard dip meter in most wells during the four sampling periods. River flow measurements were performed by a SENAMHI technician using a standard horizontal axis flow meter (SIAP, 0.05 to 5 m s^−1^) at many surface water sites in April and July 2014.

### Water Sample Site Characteristics

Of the 27 sites sampled for water quality, six were surface sites located either in the Urmiri River channel or >2 km from mining activity, tailings or ponds (Fig. 1); these are referred to as ‘mining-separated surface sites’. They include one spring (*sd*), two irrigation channels (*sa*, *se*) and three river sites (*sb, sc, sf*). Spring water from hill slopes is generally fed through plastic tubing into tanks that are covered with wooden boards. Irrigation channels and pools used to hold water are generally open and constructed of concrete and take water from rivers, streams and springs. Approximately 33% of mining-separated surface sites are understood to be used for human consumption and at least 33% are used for irrigation. All mining-separated surface sites are used for livestock watering and sometimes for domestic purposes.

Ten groundwater wells were sampled (*ga-gj*). Wells are shallow (~2–3 m deep), unlined and generally covered with a loose metal or wooden lid, but sometimes they are open. They are usually operated by hand or electrical pumps. Approximately 40% of sampled wells are understood to be used as a source of water for human consumption and 60% for irrigation purposes during the dry season; all are used for livestock watering and sometimes for domestic purposes. Two thermal waters (*ta, tb*) were also sampled at sites used for bathing.

Nine surface sites located close to or downstream of mining activity, tailings or ponds (referred to here as ‘mining-associated sites’) were sampled (*ma–mi*; Fig. 1). Mining activity occurs along the Antequera River (e.g., Totoral and Avicaya mines) and results in inputs of AMD; also there are several (historical and new) tailing heaps within and at the sides of the river. There is considerable refuse in the Antequera River, as well as little vegetation and often algae (especially in the middle reach represented by *md-mf*). Water from mining-associated sample sites is not generally directly used as a water source for irrigation purposes, but is sometimes used for domestic purposes (e.g., clothes washing) and is accessible to grazing animals.

### Laboratory Analysis

Water samples were analysed for 22 cations (Al, As, B, Ba, Ca, Cd, Co, Cr, Cu, Fe, K, Li, Mg, Mn, Mo, Na, Ni, Pb, Sb, Si, Sn, Zn) and three anions (Cl, F, SO_4_). We do not report on NO_3_ due to shipment concerns for hold times. Cations were analysed using a Varian 720-ES ICP-OES CCD Simultaneous ICP Optical Emission Spectrophotometer and anions using a Dionex (Thermo) AS50 Autosampler. Blanks were prepared and analysed following the same procedure as all field samples using ultrapure Milli-Q water. The Battle02 certified reference material and internal standards were used to test analytical accuracy and analysed concentrations were within reported ranges. Analytical precision was evaluated by analysis of duplicate samples (10% of the total samples analysed) and was < 5% for all duplicates, except for those with very low concentrations.

### Water Quality Data Handling

Element concentrations (Table S4) were assessed with reference to: (a) Bolivian class ‘A–D’ criteria for receiving waters (referred to here as ‘A’, ‘B’, ‘C’ or ‘D’ criteria) (Table S5); (b) World Health Organization (WHO) guidelines for drinking water quality (2011) (Table S5); (c) Food and Agriculture Organization (FAO UN 1985) recommendations for non-restricted use of water used in agriculture; (d) FAO recommendations for livestock. Hazard Quotients (HQs) were calculated for individual concentrations by dividing them by the respective element Bolivian ‘A’ criteria for all elements except Li (FAO recommendation).

### Development of a CWQHR

The first well known water quality index (WQI) was developed by Horton (1965), with many others having followed (e.g., CCME 2001; Cude 2001; Said et al. 2004), and which incorporate a range of biological and chemical water quality indicator parameters, as reviewed in Lumb et al. (2011). Although useful in their own right (e.g., for biological assessment) and for given localities, situations and users, there are number of issues that detract from their wide usage, such as: (i) fairly complicated index determination methods arising from the use of mathematical functions (CCME 2001) that generally require training and sometimes computer programs, and that often incur limitations or step-wise incremental increases in error (cf., Lumb et al. 2011); (ii) qualitative descriptions that are understandable by general users are required, but can be missing or ambiguous; (iii) existing WQIs are not applicable in areas affected by mining or natural contamination; (v) WQIs do not encompass all aspects of water use, including potable, agricultural, ecological and recreational uses, and broader environmental aspects.

To avoid these limitations and complications, and more specifically to provide an understandable and usable management tool for local community leaders and environmental managers on the Bolivian Altiplano (and similar environments), we have developed a qualitative CWQHR (Table S6). The main purpose of this relatively simple tool is to distinguish waters suitable for certain uses from those that are unusable, and where necessary to recommend levels of treatment to improve waters for specific uses. The CWQHR is based on water chemistry (Table S4) but also the presence of algae, suspended particulate/organic material such as animal droppings, and/or stagnation. It incorporates simple mathematics in the form of HQs and optional calculation of sodium adsorption ratios (SAR). Although WQIs generally use a percentage ranking, whereby a low percentage indicates poor quality and a high one good quality, our CWQHR uses a 1 (good quality) to 10 (highly contaminated) scoring range. The rationale for this extends to the concept of risk and the usefulness of simultaneously considering the level of social vulnerability to water issues, which is classified numerically on a scale of 0–10 (<2.5 low, >7.5 very high vulnerability; Table S3).

## Results

### Vulnerability Assessment

Table 1 provides a summary of the Vc1–Vc6 and Vt scores for each of the 13 communities *ca–cm* (based on the averaged vis of households within each community; Table S2) and for the entire study area. All surveyed communities (Vt 5.00–6.93) and therefore the entire study area (Vt 6.15) obtained Vt scores corresponding to the category of high total vulnerability to water quality and scarcity issues (Table S3). This indicates that the majority of people surveyed have the following: (i) limited access to potable water; (ii) mainly primary education only; (iii) almost no training on water issues; (iv) marginal income; (v) some level of social organisation, but no autonomy in decision making; (vi) a perception that they and/or their livestock have suffered ill-health due to water contamination (Table S3).

**Fig. 3:**
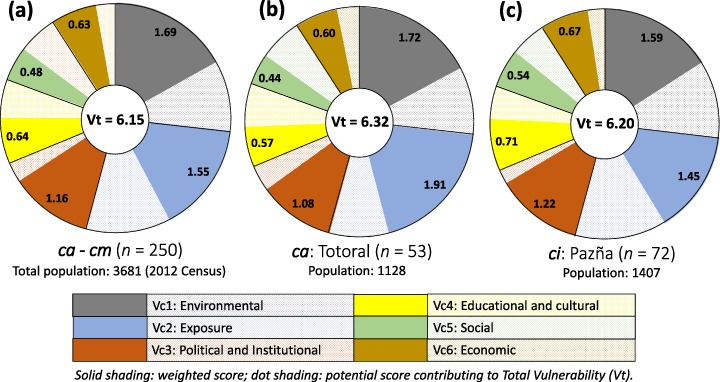
Proportional contribution of Vc 1–6 scores to the Vt for **a** the entire study area, **b** community *ca* (Totoral) and **c** community *cm* (Pazña)

For the entire study area, the political and institutional component (Vc3) was the highest overall Vc (8.05) and the social component (Vc5) the lowest (4.60) (Table 1), reflecting the relative vulnerability of these components in the study area based on the assumed weighting of vis (Tables S1 and S2). However, inclusion of all vis as weighted to Vts, as opposed to Vcs, results in the environmental component (Vc1) being proportionately higher (1.69) than other Vcs, as illustrated in Fig. 3a for the entire study area. Specific findings for each Vc are discussed in the Supplementary Information. We note that because the community level Vc and Vt scores are the average of participant households within each community, scores mask variations between households. Attention should also be paid to the populations of communities and the varying number of participant households within each community (Table 1).

### Channel Profiles and River Flows

**Fig. 4:**
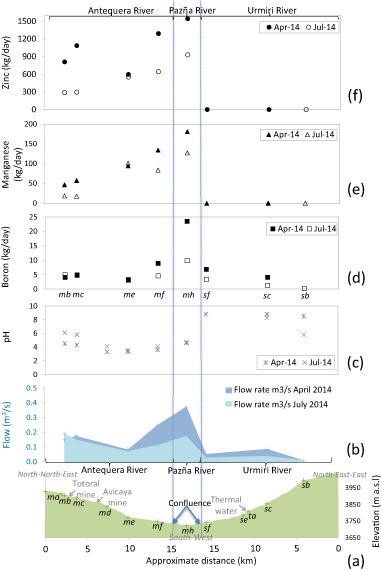
Graphs showing: **a** conceptual elevation profile (*y*-axis exaggerated by a factor of 20 relative to *x*-axis) against approximate distance from upstream to downstream of Antequera (North-north-east) and Urmiri rivers (North-east-east) with confluence at the mid-point into the Pazña River; **b** river flows (m^3^/s) during April and July 2014; **c** pH at river sites; **d** boron flux (kg/day; calculated using river flow, m^3^/s as L/d, and concentration, mg/L as kg/L); (**e** and **f**) manganese flux (kg/day) and zinc flux (kg/day) at river sites along the Antequera (*mb, mc, me, mf*), Urmiri, (*sb, sc, sf*) and Pazña (*mh*) rivers during April (end of wet season) and July (dry season) 2014. See Fig. 1 for birds-eye view of sample sites and rivers

River channel profiles provide a useful basis for presenting river flow and chemical data. This is achieved using a schematic of the elevation cross-section with distance from upstream sample sites on the Antequera and Urmiri rivers to sample sites after these rivers meet to form the Pazña River, and the locations of mines and thermal waters are also included (Fig. 4a). Figure 4b shows the river flow along these channel sections during April 2014 (end of wet season; e.g., 0.18 m^3^/s at *mc*) and July 2014 (dry season; e.g., 0.15 m^3^/s at *mc*). Spatially, flows increase in the rainy season from upstream Antequera to the lower reach (e.g., for April 0.18 m^3^/s upstream at *mc* to 0.25 m^3^/s downstream at *mf*) due to rainfall, runoff and tributary inputs. By contrast, the river experiences losses from upper to lower reaches in the dry season (e.g., in July 0.15 m^3^/s at *mc* to 0.11 m^3^/s at *mf*). In comparison, the Urmiri River generally has a relatively low flow (mid-stream at *sc* is 0.08 m^3^/s in April and 0.04 m^3^/s in July). The Antequera contributes >70% of flow in the Pazña River and the Urmiri ~15%.

### Water Quality

Chemical data show the clear chemical differences between the Antequera and Urmiri rivers and how these impact the Pazña River. For example, Fig. 4c illustrates the generally alkaline conditions (pH > 7) of the Urmiri River (e.g., *sc*) and acidity (pH ~4) of the Antequera River (e.g., *mf*). Together with Fig. 4b, this suggests that the more significant volumetric contribution of the Antequera River results in the acidification of the Pazña River (e.g., *mh*). The acidity has implications for the transport of metals and metalloids because it promotes the dissolved fraction (Stumm and Morgan 1996). This is illustrated by Figs. 4f and e, which show how high fluxes of Zn (800–1500 kg/day) and Mn (~100 kg/day), largely sourced from the upstream Antequera, are transferred along the Pazña River (e.g., *mh*). The near zero input of these and other mine related metals and metalloids from the Urmiri demonstrate the huge impact of mining on the Antequera, and also the natural background state of the rivers.

**Fig. 5:**
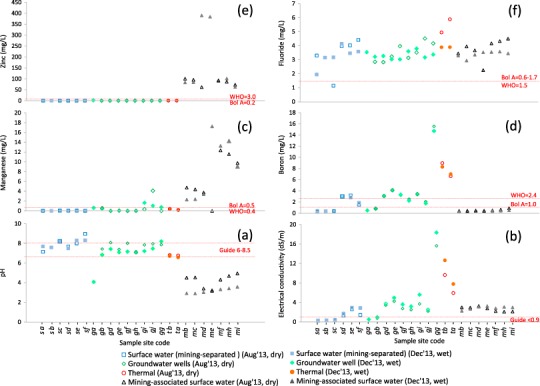
Graphs showing dry (August 2013, empty symbols) and wet (December 2013, *solid symbols*) season conditions at mining-separated surface sites (*sa–sf*; *blue squares*), groundwater wells (*ga–gj*; *green diamonds*), thermal sites (*ta, tb*; *red circles*) and main channel Antequera and Pazña River mining-associated sites (*mb–mf*; black triangles) for **a** pH, **b** electrical conductivity (dS/m), **c** manganese (mg/L), **d** boron (mg/L), **e** zinc (mg/L) and **f** fluoride (mg/L). *Dashed lines* indicate Bolivian A (‘Bol A’) and WHO (2011) guidelines for recommended pH range and maximum concentration of elements and for general recommended limit for electrical conductivity in drinking water (<0.9 dS/m)

Overall, the water quality data clearly show how mining-associated sites (*mb - mi*) are characterised by acidic pH (Fig. 5a) and very high concentrations of many metals, e.g., Zn (Fig. 5e) and Mn (Fig. 5c). Our findings of mining related contamination support previous studies that reported the Antequera River as a main contributor of contamination to Lake Poopó (Navarro Torres et al. 2012) and the region (Quintanilla et al. 2012; García et al. 2010; Ramos Ramos 2014). The characteristics of these waters are similar to those in other mining-impacted areas of Bolivia, such as those around Potosí and in the Río Pilcomayo that are affected by discharge of wastes from extraction and processing of the Cerro Rico de Potosí ores (Hudson-Edwards et al. 2001; Strosnider et al. 2011, 2014).

The Urmiri River at *sb* and *sc* is contaminated by elements such as F (Fig. 5f) and Sb, with similar fluxes occurring in the Antequera River (*mb-md*). The majority of wells in the study area (Fig. 5, sites *gd-gg*) have relatively high natural F in addition to high salt content (EC, Fig. 5b) and elevated B (Fig. 5d), which also appear to be naturally sourced, as supported by the high concentrations found in thermal waters (*circles* in Fig. 5). These findings agree with those of García et al. (2010) and Quintanilla et al. (2012) in the study area and of Tapia et al. (2012) in the area north of Lake Poopó, and of Hudson-Edwards and Archer (2012) in the Argentinian Andes.

### CWQHR assessment

**Fig. 6:**
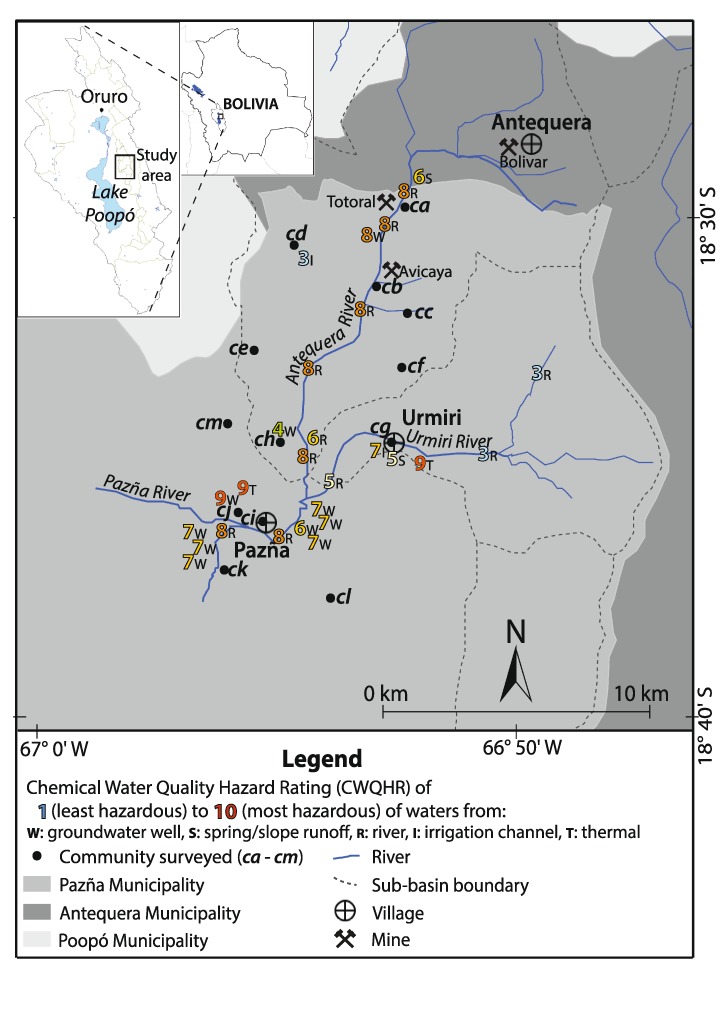
Map of study area showing Chemical Water Quality Hazard Index (CWQHR 1–10; least–most hazardous) for sites in 2013–2014 (see Table S6 for CWQHR description, and Fig. 1 for site codes) and communities involved in the vulnerability survey (all determined to be high total vulnerability, Vt 5.0–7.5, Table S3). Inset map shows location of study area within Lake Poopó Basin and Bolivia

The CWQHRs have been calculated using the chemical datasets of sample waters (Table S4; Fig. 6). Of the 27 water quality sites assessed, 22% were classified as CWQHR 3, 4 or 5 and of good to fair chemical quality. Three sites were assessed as CWQHR 3 (*sa, sb, sc*; Tables S5 and S6; Figs. 1 and 4) and hence of being of relatively good chemical water quality, although there is a health-risk caution over elevated F. CWQHR 3 rated waters came from two sites in the upstream section of the Urmiri River (*sb, sc*; Fig. 1) that are available for livestock watering and may feed into sources used for human consumption (e.g., community *cg*), and an irrigation and livestock watering channel (*sa*, in community *cd*). The relatively good quality of the upstream Urmiri River sites (*sb, sc*) can be illustrated by comparison of pH, Mn and Zn with that in downstream river samples (*mh* and *mi*; Fig. 5a, c and e).

The CWQHR 4 water came from a well (*gb*) near community *ch*, for which EC was at the upper level of recommendations for drinking water (~0.9 dS/m) and which had a few samples with Fe and Mn just over Bolivian ‘A’ criteria. Waters at two sites were classified as CWQHR 5 (Table S6) and they came from a spring (*sd*, near community *cg* used for human consumption; Table 2) and in the downstream Urmiri River (*sf*, livestock watering). In addition to the exceedance of several health-risk elements (F, Sb or As), the CWQHR 5 sources also had elevated B and salt content (EC 1–3 dS/m), which renders them unsuitable for human consumption and not recommended for irrigation of lower tolerant crops.

**Table 2:** Example site water chemical data and Chemical Water Quality Hazard Rating (CWQHR, Tables S4 and S6) for some of the locations in Fig. 1 during dry (August 2013) and wet (December 2013) seasons (rainfall in Fig. S1)

	*Site code:*		*sc*		*sf*		*se*		*gj*		*mc*		*mi*		*Ta*	
	Type:		River		Spring/slope		Irrigation canal		Well		River (near mines)		River (downstream)		Thermal	
Parameter (mg/L except pH, EC, SAR)	Bolivian ‘A’ criteria	CWQHR	3		5		7		7		8		8		9	
			Dry	Wet	Dry	Wet	Dry	Wet	Dry	Wet	Dry	Wet	Dry	Wet	Dry	Wet
pH	6.0 –8.5		8.2	8.2	7.7	7.5	8.0	8.3	8.0	7.5	4.5	2.9	5.0	3.6	6.8	6.6
Electrical Conductivity (EC, dS/m)	<1.5^a^		0.30	0.39	1.3	1.7	2.6	2.9	2.2	2.5	2.9	2.7	2.1	3.0	5.9	7.8
Sodium Adsorption Ratio (SAR)^b^	–		0.8	1.9	7.9	7.4	15	29	5.9	5.2	0.9	1.2	1.7	1.7	30	60
Total Dissolved Solids (TDS)	1000		150	192	653	850	1300	1470	1100	1260	1470	1330	1060	1500	2960	3920
Cl, chloride	250		12	12	229	212	605	551	285	238	217	62	226	127	1613	1634
F, fluoride	0.6–1.7		1.2	3.2	4.0	4.1	4.0	3.5	4.5	3.2	4.0	2.9	4.5	3.5	5.9	3.9
SO_4_, sulphate	300		54	47	74	74	45	46	651	480	1680	1050	944	1080	30	30
Al, aluminium	0.2		<0.0312	<0.0312	<0.0312	<0.0312	<0.0312	<0.0312	<0.0312	<0.0312	16.1	29.7	13.3	16.9	<0.0312	0.03
As, arsenic	0.05		<0.0248	<0.0248	0.027	<0.0248	<0.0248	<0.0248	0.126	0.105	0.048	0.120	<0.0248	<0.0248	<0.0248	<0.0248
B, boron	1.0		0.36	0.36	3.0	3.0	3.2	2.8	2.1	1.7	0.37	0.42	0.87	0.50	6.6	7.0
Cd, cadmium	0.005		<0.0011	0.002	<0.0011	<0.0011	<0.0011	<0.0011	<0.0011	<0.0011	0.43	0.36	0.23	0.28	<0.0011	<0.0011
Cu, copper	0.05		<0.0044	<0.0044	<0.0044	<0.0044	<0.0044	<0.0044	0.007	<0.0044	0.12	0.32	0.32	0.52	<0.0044	<0.0044
Fe, iron	0.3		0.035	0.049	0.003	0.22	0.032	0.026	0.098	0.033	0.76	92	0.27	1.8	0.58	0.40
Li, lithium	2.5^c^		<0.029	<0.029	1.6	1.8	3.3	3.3	1.7	1.6	0.16	0.09	0.67	0.43	9.8	9.0
Mg, magnesium		100	10	12	17	19	10	12	25	23	21	20	29	29	11	13
Mn, manganese	0.5		0.030	0.072	0.022	0.035	0.070	0.033	4.1	1.0	4.4	2.4	9.8	9.1	0.17	0.18
Na, sodium	200		18	48	220	220	440	850	310	240	84	74	120	130	1100	2200
Ni, nickel	0.05		<0.0074	<0.0074	<0.0074	<0.0074	<0.0074	<0.0074	<0.0074	<0.0074	0.085	0.11	0.15	0.15	<0.0074	<0.0074
Pb, lead	0.05		<0.0251	<0.0251	<0.0251	<0.0251	<0.0251	<0.0251	<0.0251	<0.0251	<0.0251	0.083	<0.0251	<0.0251	<0.0251	0.036
Sb, antimony	0.01		<0.0192	<0.0192	0.039	<0.0192	0.040	0.020	<0.0192	<0.0192	0.034	<0.0192	0.023	<0.0192	<0.0192	0.042
Sn, tin	0.025^d^		<0.0353	0.043	<0.0353	0.060	<0.0353	0.093	0.040	0.062	<0.0353	0.046	0.051	0.095	0.038	<0.0353
Zn, zinc	0.2		<0.0026	<0.0026	0.030	<0.0026	0.003	<0.0026	0.046	0.033	95	85	73	63	0.006	0.014

< Denotes below calculated method detection limit

^a^ Generally, recommended drinking water EC < 0.9 dS/m (<600 p.p.m. TDS) and maximum 1.5 dS/m (1000 p.p.m. TDS; recommended by the WHO (2011) for taste and palatability)

^b^ SAR = [Na meq/l]/({[Ca meq/l] + [Mg meq/l])/2})^1/2^

^c^ FAO UN (1985) recommendation for non-restricted irrigation use

^d^ UK Environment Agency non-statutory guideline for the protection of aquatic life (surface waters)

When the CWQHR exceeds 5 notable concerns in water quality begin to appear due to higher frequencies or magnitudes of chemicals exceeding Bolivian ‘A’ criteria and in many cases elevated EC (Fig. 5b). Twenty-one of the 27 sites sampled yielded waters of CWQHR 6–10, which indicates they are unsuitable for human consumption without treatment and are not recommended for irrigation (or livestock watering in many cases). This included 90% of sampled groundwater wells, 67% sampled surface waters and all sampled thermal waters, as detailed below.

Three sites were classified as CWQHR 6 (Fig. 6, Table S6) and these were from a farm well (*gc*), a spring/slope runoff (*ma*, near Totoral mine and *ca*) and from a weir on a tributary into the Antequera River (*mg*) (Figs. 1 and 5). Waters at all sites are available for livestock watering and domestic use, and may in some cases contribute to water used for human consumption. In addition to the exceedance of Bolivian ‘A’ criteria for health-risk elements (F, Sb and in some cases As and Cd) that may be naturally sourced, concern is heightened due to at least one significantly elevated element that may be linked to mine water migration or infiltration (e.g., Zn, Ni, Fe or SO_4_).

Waters at seven sites were classified as CWQHR 7 (Fig. 6, Table S6) and these came from an irrigation channel (*se*, in community *cg*) and six wells (*gd, ge, gf, gh, gi*, and *gj*-east of community *ci* and near community *ck*) (Figs. 1 and 5, see Table 2 for example of *se* and *gj*). The irrigation channel (*se*) is used both for irrigation and domestic purposes, and has high EC (~3 dS/m), Na, Cl, B, Li, F and, sometimes, Sb. The wells *gd, ge* and *gf* are understood to be used for human consumption, livestock watering and irrigation. Samples from these wells had high EC (2–7 dS/m), Na, Cl, B, Li and F. The *gh*, *gi* and *gj* wells are supposedly used for irrigation in the dry season, and samples had high EC (2–6 dS/m), Na, SO_4_, F, B, As and, sometimes, Sb. Also, *gi* and *gj* have high Mn, and *gi* sometimes high Li. Particular concern for all of these wells relates to exceedance of Bolivian ‘A’ criteria for health-risk elements F, Sb and As, which appear to be of natural origin.

Eight sites were categorised as CWQHR 8 (Fig. 6, Table S6) and appear to have been significantly affected by mining, as they contain high SO_4_ and very high concentrations of many metals and metalloids often orders of magnitude above Bolivian ‘A’ guidelines: (i) Antequera River sites (*ga, mb, mc, md, me* and *mf*) with extremely high concentrations and fluxes of Zn (Figs. 4e and 5c), Fe, Al and Cd (HQs > 100), and high EC (Fig. 5b) and Mn (Figs 4e and 5c), Cu, As, Ni, Sb, F (Fig. 5f), Pb and SO_4_ concentrations (see Table 2 for example of *mc*); (ii) the continuation of the Antequera River to sites on the Pazña River (*mh* and *mi*), with extremely high concentrations and fluxes of Zn (Figs. 4f and 5e), Al, Cd and Mn (Figs. 4e and 5c), and high SO_4_, Cu, Fe, Ni, Sb and Co (see Table 2 for example of *mi*). Although rivers are not generally directly used as water sources other than for domestic purposes (e.g., clothes washing), livestock watering does occur and hence food-chain impacts may be a concern. The rivers may carry contamination significant distances downstream and thus there is also concern over infiltration into groundwater (e.g., as suggested by Mn in *gi* and *gj* wells; Fig. 5c) and possible tributary inputs to Lake Poopó or the lake bed when dry.

Sites classified as CWQHR 9 were for the two thermal waters (*ta* near *cg*, and *tb* near *cj*) and one well site (*gg* near *cj*) (Fig. 6, Table S6). These waters all exhibited a similar chemical signature of extremely high EC (Fig. 5b), Na and Cl, and very high B (Fig. 5d), high Li, F (Fig. 5f) and Sb, and sometimes high Fe (see Table 2 for example of *ta*). Thermal waters (see Fig. 5) are known to contain naturally elevated concentrations of elements of health significance (e.g., F, B, and Sb) (Stauffer and Thompson 1984; Hudson-Edwards and Archer 2012).

## Discussion

### Use of Vulnerability and Water Quality Assessments to Prioritise Areas for Assistance

Population centres that were assessed to be of the poorest water quality can be regarded as priority areas for assistance. With this in mind, the discussion here focuses on data from the two highest population centres, *ca* and *ci*, whose rivers were assessed as CWQHR 8, as a means of drawing together the main findings of the vulnerability and water quality assessments.

Half of the surveyed population (125/250 people) were from *ca*, Totoral (53 surveyed out of 1128 people), and *ci*, Pazña (72 surveyed out of 1407 people). These communities collectively represent 69% of the total population of the study area communities (2535/3681) and 43% of the population of the municipality (2535/5853) and live in an environment characterised by waters unacceptable for human consumption (and often for livestock and irrigation) without significant treatment (Fig. 6); 21 of the 27 sites sampled yielded waters of CWQHR 6–10, including 90% of sampled groundwater wells, 67% of sampled surface waters and all sampled thermal waters.

Communities *ca* and *ci* and the entire study area yielded Vt scores (Vt 6.32, 6.20 and 6.15, respectively) corresponding to high total vulnerability to water quality and scarcity (Fig. 3, Table S3). For the entire study area the weighted environmental (Vc1) score of 1.69 contributed the highest to the Vt, reflecting in particular the large number of households with insufficient water available for personal use (average 13 vs. 20 L/c/d minimum WHO recommendation), livestock and/or irrigation. This was especially the case for *ca* as this community’s Vc1 score was above the averaged study area score (Table 1), which was also due to people’s perception of water contamination being responsible for illness in humans and animals (*cg*, Fig.1 and vi5 in Table S2) and because many surveyed households do not have access to the piped network and rely solely on untreated water (e.g., from springs, such as *sd*). The findings of the chemical water quality assessment support residents’ concern over the quality of water in their environment, although many of the reported human health concerns (e.g., gastrointestinal) may be bacteria related and warrant investigation. Rivers and groundwater in communities close to and downstream of mining activity are characterised by mine-related (e.g., Zn, Al, Mn, Fe, Cd, SO_4_ etc., as in Fig. 5c and e) and natural contamination (e.g., F, B, Sb, high salt content; Fig. 5b, d and f) above Bolivian ‘A’ and WHO guidelines. Although some populations who rely on water from upper catchment areas (e.g., *cd*) that is of relatively good chemical quality, some rivers, irrigation channels and springs near communities unaffected by mine activity (e.g., *cg*, *sf, se, sd*) are also found to have natural contamination at levels above guidelines (e.g., Fig. 5f), possibly due to the influence of local thermal waters (e.g., *ta*).

Also problematic for the area is the dependence of people on rainwater during the rainy season, as this limits the amount available for crop production, which necessitates the use of naturally saline irrigation waters that potentially contain elevated metals and metalloids (e.g., *se*, *gh-gj*). This in turn has implications for the economy of the area, including the way people work (i.e., irregular, unguaranteed income based on seasonal shifts between agriculture and mining) and high migration. Furthermore, uptake of metals and metalloids by livestock watering also represents an issue for agricultural production.

Figure 3 shows the weighted Vc2 (exposure) contribution to the study area and communities’ *ca* and *ci* Vts (Table 1, Fig. 1). Community *ca* obtained the highest weighted Vc2 score and is therefore most vulnerable with respect to the exposure component. This is primarily due to the fact that *ca* is a high population mining community and is <500 m from the Antequera River and main mine workings (CWQHR 8; Fig. 6, sites *mb-mc*). In contrast, *ci* obtained a weighted score below the study area average (Fig. 3). This was due to its being > 2 km downstream of main mining activity (Fig. 1) and >500 m away from a river assessed as CWQHR 8 (*mh-mi*), and also because surveyed residents are on average fewer per household, travel less than the study area average for water and have both lower than average number of livestock and area of cultivated land being exposed than the study area average (Table S2). However, *ci* is still <1000 m from the Pazña River (*mh-mi*), also assessed as CWQHR 8 (Fig. 6), in addition to having access to groundwater wells determined as unfit for most purposes without treatment. As such, people (and their animals) living along the entire Antequera and Pazña river reaches remain highly vulnerable to mining-related and natural contamination (Figs. 4 and 5). Of particular concern is the potential for migration of contaminants into groundwater for all communities close to and downstream of mining activity (e.g., Mn in *gi* and *gj* groundwater wells; Fig. 5c).

The weighted political and institutional component contribution (Vc3 1.16, Fig. 3a) to the study area Vt indicates the importance of related topics to residents. Of particular concern is the population centre of *ci*, where averaged surveyed households considered (i) themselves to have no influence on water related decision making, (ii) their community to be poorly organised, and (iii) their community to have no relationship with mining operators or the municipality. This suggests that local residents feel powerless to tackle the issues relating to, for example, the contamination of the Pazña River (*mh-mi*) but also to scarcity issues, which has become a dominant issue in 2016 following the drying out of Lake Poopó (National Geographic 2016), and illustrates residents’ very real concerns and doubts over the restoration and preservation of their environment. This is also true of *ca*, the main difference from *ci* being that surveyed *ca* residents considered themselves fairly well organised and, as detailed below, that *ca* residents were also determined as less vulnerable in terms of educational, social and economic components than surveyed *ci* residents.

Although educational and cultural (Vc4 0.64; Fig. 3a, Table 1), social (Vc5 0.48) and economic (Vc6 0.63) components were lesser contributors to the study area Vt, numerous aspects stand out from the indicators used to determine these Vcs, including for Vc4, lack of training on water issues (vi21), for Vc5, high migration (vi22), and for Vc6, diversification of production (crops mainly for self-consumption, vi27) and high poverty level (vi28).

In *ci*, where in addition to <5% of averaged surveyed households having a family member with training on water issues, levels of illiteracy are higher than in *ca* and the study area average. Therefore, the overall Vc4 score for *ci* is also higher than *ca* and the study area average. Also, *ci* households had a much higher migration score (vi22) than *ca*, possibly relating to different dominant employment sectors; this is reflected in Vc6, whereby the main economic activity of *ci* surveyed households was on average dominantly (seasonal) agricultural work, versus mining and technical roles in *ca* (vi26, Table S2). Both communities produce crops mainly for self-consumption as opposed to selling them, and for the entire study area are dominantly ranked as poor (Census 2012). Hence, *ci* obtained Vc3, Vc4, Vc5 and Vc6 scores that were higher than for *ca* and the study area average, whereas *ca* scored below average on these Vcs, but higher than *ci* and the study area average on Vc1 and Vc2, reflecting the different vulnerabilities of these communities. As a mining centre, the vulnerability of *ca* is characterised by above average scores in environmental and exposure elements, whereas the downstream vulnerability of *ci* is characterised by above average political, educational, social and economic factors.

### Recommendations for Management and Remediation

Of the 27 water sample sites assessed in the Bolivian Lake Poopó Basin, 78% were classified as CWQHR ≥ 6 and therefore unsuitable for human consumption without significant treatment due to high levels of metals and/or salt; 90% of groundwater sites, 67% of surface water sites, and 100% of thermal water sites fell into this category. Many of these waters are also not recommended for irrigation or livestock watering. Natural contamination is characterised by high concentrations of salts and exceedance of Bolivian ‘A’ criteria for some elements of health significance (e.g., F, B and Sb). Very high concentrations of mining-related contaminants (e.g., Zn, Al, Mn, Fe, SO_4_ and Cd) make many waters unsuitable for any use without significant treatment, and this represents a considerable environmental problem.

Surveyed households in the study area were on average found to be highly vulnerable to water hazards, with restricted access to basic water related services and only sufficient quantities of water to meet basic health requirements. The majority of surveyed people only complete a basic level of education and few have received training on water issues. Additionally, income levels are generally very low and as a result human and animal health may be at risk from insufficient water and/or poor water quality. Our findings demonstrate an urgent need to improve access to water and to ensure that the water available is safe enough for various end-users and intended purposes, including human and animal consumption and irrigation for agriculture.

Overall, the severity of the levels of natural and mine contamination in many waters in the region demonstrate the need for compliance with water quality legislation and for stakeholders to take increased action on their water related and environmental responsibilities. In the more immediate future, priorities might therefore include the following: placing use restrictions on sites assessed as CWQHR ≥ 5; routinely assessing bacterial quality; taking steps to protect water sources against further contamination; exploring for additional sources of fair to good chemical quality water; expanding water harvesting and conservation; and improving and maintaining basic water treatment, storage and supply infrastructure. Desalination treatment and mine waste and water remediation on the other hand are options for the longer term that would be both challenging and require substantial investment.

When considering the CWQHR of assessed sites (Fig. 6, Table S6), two main areas stand out as priority importance for intervention for increasing the supply of potable water as well as livestock and irrigation waters. The first area is that in close proximity to the Pazña River (CWQHR 8) and floodplain wells (CWQHR 7) and includes Santa Filomena (*ck*), Pazña (*ci*; high population centre) and San Martín (*cj*). The second area includes communities near the Antequera River (CWQHR 8), such as Totoral (*ca*; high population centre) and Avicaya (*cb*). Given that we find the Antequera River to be highly contaminated by metals and metalloids and that we assess the communities as highly vulnerable, we recommend the undertaking of a detailed groundwater study to assess any related contamination and contaminant pathways. In addition, given that livestock (that often later enter the food-chain) openly graze and frequent the river for watering, we also highlight the need for investigations relating to food-chain impacts and possibly health risk in animals and people.

## Conclusions

This study has presented methods developed through an innovative academic-NGO-community collaboration to assess hazards and vulnerabilities linked to water availability and quality. The results produced exemplify how such assessments can yield information that has the potential to generate positive change for communities that lack sufficient water of safe quality for maintaining human, animal and ecosystem health. Such communities are many on the Altiplano and within the Andean mining belt (e.g., Miller and Villarroel 2011; Perreault 2012; Rojas and Vandecasteele 2007; Salvarredy-Aranguren et al. 2008) and may benefit from assessment using our methodological framework. Likewise, the methodology could also be applied to benefit similar situations and environments elsewhere, for example, the Río Tapajós (Brazil; Barbien and Gardon 2009), the Mekong River (Cambodia; Berg et al. 2007) and the Odiel catchment (Spain; Sánchez España et al. 2005), where communities may require assessment and assistance regarding vulnerability to natural or mine related contamination of their water and environment. Undertaking of this type of assessment could also help mitigate environmental disasters and contamination related to, for example, mine dam failings (e.g., Río Doce, Brazil, November 2015; WISE 2016).

General responsibilities of specific stakeholders that could improve access to water and help ensure that water is safe enough for various purposes are highlighted as follows. Ideally, regional authorities (e.g., in our study, the Government of Oruro) should meet legal requirements to prevent contamination of water bodies; in addition, they should: identify, monitor and manage major sources of contamination; undertake risk assessments of water resources (and the food-chain) and implement appropriate management strategies, including monitoring and chemical and bacterial certification of water sources; improve and maintain basic water supply infrastructure and expand water collection, conservation and treatment systems through regional investment in appropriate infrastructure. Municipalities (e.g., local authorities) should meet legal requirements to identify and manage sources of contamination (including monitoring and controlling wastewater discharges) and undertake periodic chemical and bacterial monitoring of potable water sources and build and operate water and wastewater treatment facilities. Communities should have individuals who are informed and trained in collecting, analysing and interpreting water quality data and form river-basin management committees with the other stakeholders. Finally, mining cooperatives and companies should build infrastructure to adequately and safely contain and treat mine waste and water, monitor environmental discharges and make information publicly available and adhere to national or international legislation for discharges to receiving waters.

## Electronic supplementary material

Supplementary Figure 1

Supplementary Figure 2

Supplementary Figure 3

Supplementary Information

## Footnotes

1 Pazña Municipality is well known for their cattle production, which is part of Asociación de Ganaderos del Altiplano al Desarrollo (ASAGALDE).
