# Supplementary figures and images for: Community exposure and vulnerability to water quality and availability: a case study in the mining-affected Pazña Municipality, Lake Poopó Basin, Bolivian Altiplano
Source: Environ Manage. 2017 Jun 8;60(4):555–73. doi: 10.1007/s00267-017-0893-5 (PMC5602086; doi:10.1007/s00267-017-0893-5)

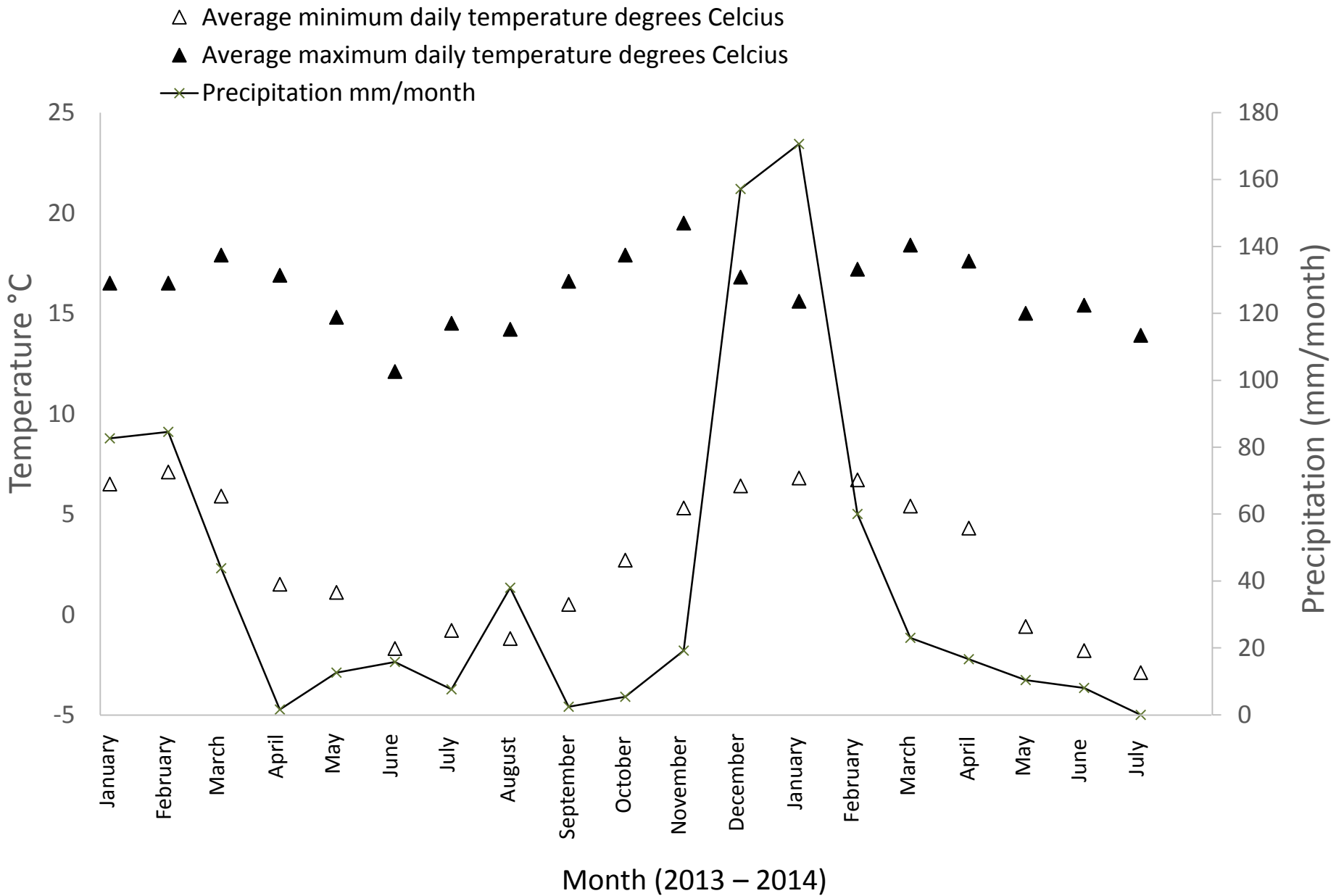

Supplement: Supplementary file 1 — Supplementary Figure 1 [file 267_2017_893_MOESM1_ESM.pdf]

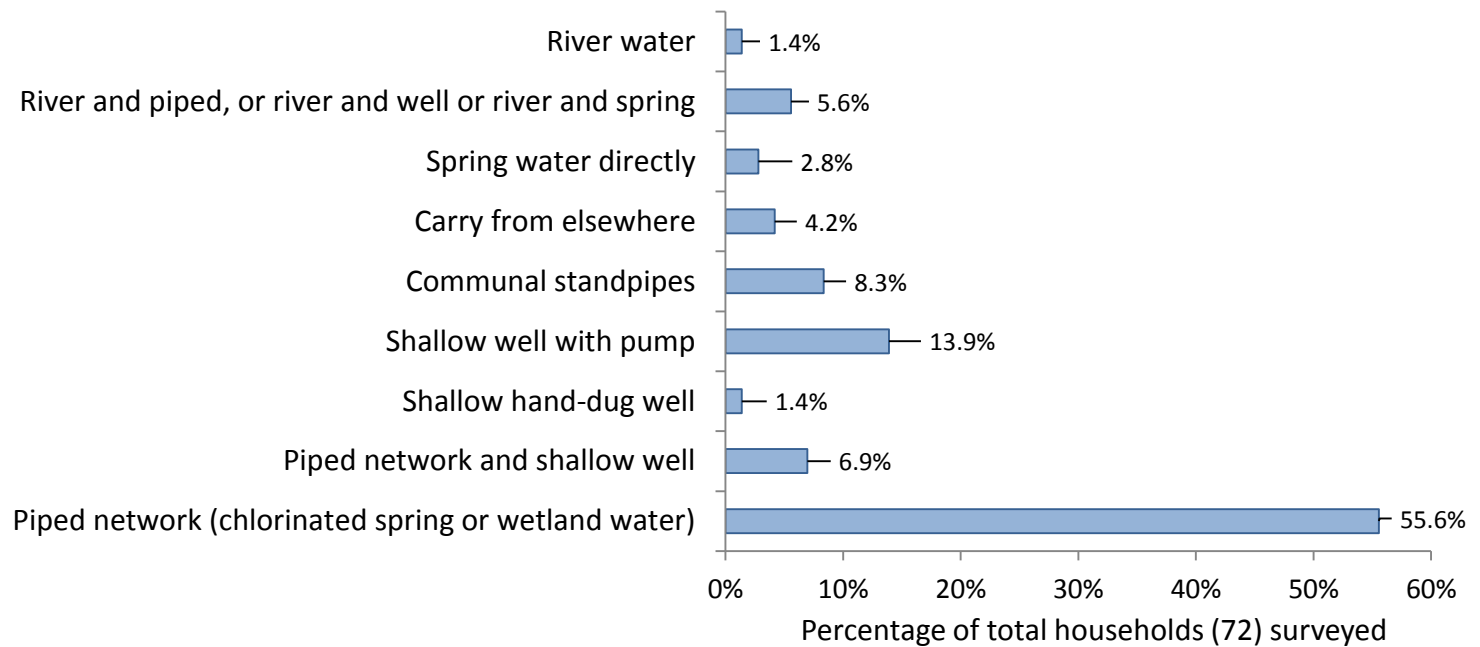

Supplement: Supplementary file 2 — Supplementary Figure 2 [file 267_2017_893_MOESM2_ESM.pdf]

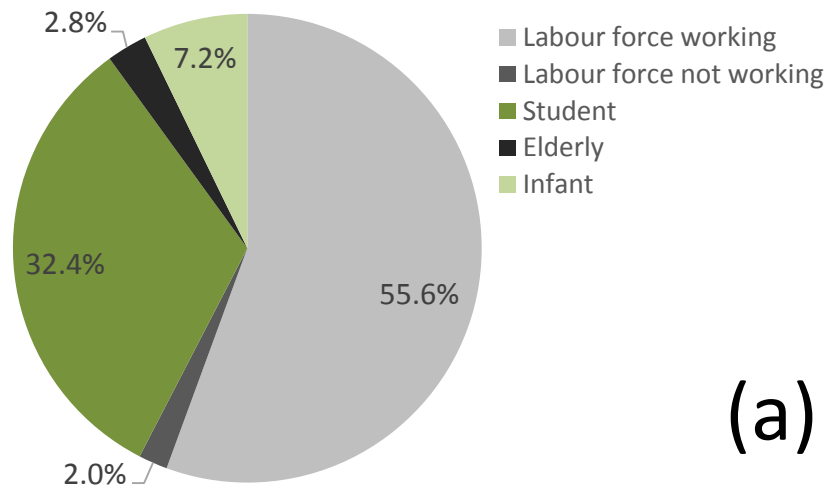

(a)

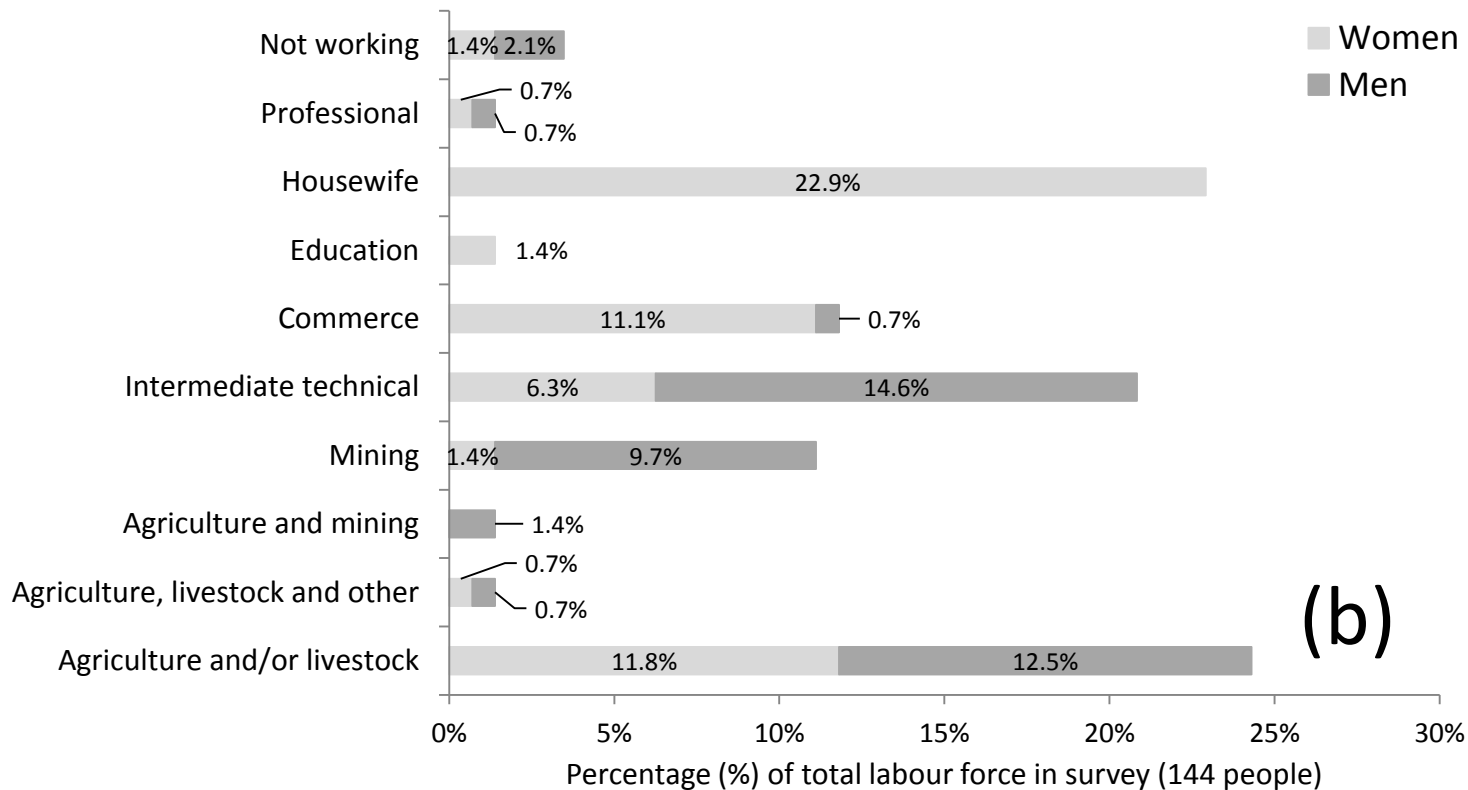

(b)

Supplement: Supplementary file 3 — Supplementary Figure 3 [file 267_2017_893_MOESM3_ESM.pdf]
