# Supplementary material for: Community exposure and vulnerability to water quality and availability: a case study in the mining-affected Pazña Municipality, Lake Poopó Basin, Bolivian Altiplano
Source: Environ Manage. 2017 Jun 8;60(4):555–73. doi: 10.1007/s00267-017-0893-5 (PMC5602086; doi:10.1007/s00267-017-0893-5)
Supplement: Supplementary file 4 — Supplementary Information [file 267_2017_893_MOESM4_ESM.docx]

**SUPPLEMENTARY INFORMATION**

## **S1. Definition of vulnerability types towards the risk of water quality and scarcity**

Details and definitions (for this study) of the Vcs contributing to Vt are as follows.

Vc1) Environmental vulnerability is defined here as the inherent vulnerability of all living beings, as determined by their basic and productive requirements. It also relates to environmental damage (such as water contamination) (INDECI 2006). The Vc1 score assigned to a household depends on, for example, the water source used for human consumption (vi1) and their water usage (vi4).

Vc2) Vulnerability due to exposure to water issues includes population and property aspects. For example, households that are proximate to contaminated surface waters, and/or households with sensitive groups (infants <5 years, children 5-14 years, the elderly, people with disabilities and single parents), will obtain higher vi10 and vi7 scores, respectively.

Vc3) Political vulnerability is defined here by the degree of autonomy and level of influence over policy decision-making that a community has to manage risk (INDECI 2006). For example, a household within a community that is capable of influencing political decisions will obtain a lower vi14 score as they are more likely to define actions and respond to a threat.

Vc4) Educational and cultural vulnerability refers to the access to education and training programs that are concerned with the use and quality of water. For example, community members that have received training in water issues (vi20) are regarded as having a degree of foresight that improves their ability to consider and handle water issues and hence assist their community.

Vc5) Social vulnerability considers the type and level of social organisation and the degree of community participation (INDECI 2006). A well-organised community where residents participate in meetings involving water issues will, for example, obtain a low score for vi22.

Vc6) Economic vulnerability is considered here as the access to economic assets that a given population have (i.e., land, infrastructure, services and employment etc.), which is reflected in their ability to face a disaster (INDECI 2006). Households with low income levels constitute the most vulnerable sector of society with respect to this component and obtain high vi25-27 scores as they may lack the opportunity and/or ability to respond and recover from water scarcity and contamination (INDECI 2006).

**S1.1. Environmental vulnerability (Vc1; vi1-vi5)**

The vis contributing to the environmental component, Vc1, are illustrated in Fig. 2; vi1 – vi5 (Table S2). Table 1 shows that the averaged Vc1 score for the study area was 6.31 (community score range 5.18 – 8.90), and as mentioned previously Fig. 3a shows that the weighted Vc1 (1.69) was the largest contributor to the Vt score for the study area. The communities with the highest Vc1 scores, and hence most vulnerable with respect to the environmental component, were *cf*, *ch*, and *ck* (Table 1, Fig. 1), partly because most households in these communities have little water available for personal use (< 20 L/c/d) and perceive water contamination to be the source of illness in humans and/or animals.

The main contributors to the Vc1 scoring are vi1 - vi3 that refer to water sources. For vi1 and human water sources, Fig. S2 illustrates that 55.6% of surveyed households use water solely from the piped network and 12.5% use piped network water in conjunction with other sources. Piped water is not, however, necessarily available on demand, especially in the dry season. Households that do not have access to the piped network obtain (supposedly untreated) water either from wells (with/without a pump; 13.9%, 1.4%), communal standpipes (8.3%), elsewhere by carrying (4.2%), springs directly (2.8%), or solely from rivers (1.4%) (Fig. S2).

For vi2, livestock water sources, cattle reportedly largely consume water from rivers (28.6%), springs (14.3%), wells (14.3%), or *vigiñas* (manually drilled/dug shallow ponds that are used for livestock watering; 9.5%). More than half of surveyed households consider there to be insufficient water for livestock.

Results for vi3 showed that most irrigation is rain-fed during the wet season (81% of households that cultivate), although 19% of households that attempt to grow crops stated that they do not have sufficient water for irrigation and many households that do not cultivate state lack of water as the primary reason.

Low water availability is a key issue for many residents own use despite the fact that over half of the surveyed households have access to the piped network; human water usage (for consumption and to meet basic personal and food hygiene needs; vi4) was on average 13 L/c/d due to lack of availability. This is considerably less than the 20 L/c/d minimum recommended by the WHO (2003), resulting in the majority of households obtaining a high vi4 score. Within the average usage, 42% of the surveyed households had <5 L/c/d, which we classify as ‘no access’ (WHO 2003) for meeting basic water needs (i.e., consumption needs cannot be assured; hygiene is not possible unless practised at source), and suggests that exposure to potential associated health risks may be very high. Of the remainder, 43% of surveyed households had a maximum of 20 L/c/d and are classified as having ‘basic access’ (i.e., consumption should be assured; hand-washing and basic food hygiene possible; laundry/bathing difficult to assure unless carried out at source). Only 15% of the surveyed households had ‘intermediate access’ (i.e., average ~50 L/c/d; consumption assured; basic personal and food hygiene assured; laundry and bathing should be assured; WHO 2003) and thus obtained lower vi4 scores than the previous groupings. No households had optimal access (>100 L/c/d).

Further to the potential risks to health and food production associated with low water availability, the quality of the water available can also pose a potential risk, as represented by vi5. More than 55% of the surveyed population perceived that their health and/or that of their animals had been adversely affected by i) the quality of water consumed, and/or ii) food-chain effects due to irrigated (or flooded) agricultural land. For the human population, 42% made reference to gastric, dermatological and neurological conditions, and 48% of respondents stated that they believed that their livestock had become sick or had died as a result of consuming poor quality water.

**S1.2. Exposure (Vc2; vi6 – vi14)**

Figure 2 illustrates the vis contributing to the exposure component, Vc2; vi6 – vi14. The averaged Vc2 score for the study area was 5.64 (Table 1; community score range 3.57 – 7.11), with Fig. 3 illustrating the weighted score (1.55) contribution to the study area Vt. The communities with the highest Vc2 scores, and hence most vulnerable with respect to the exposure component, were *cb* and *ca* (Table 1, Fig. 1), largely due to the fact that they are high population mining centres on the Antequera River.

Scoring for Vc2 includes that of vi6 and vi8, with higher vi scores attributed to households located in communities with a relatively large population and/or with high household density, respectively. The highest populations locations are Pazña town (*ci*), Totoral (*ca*), and (*cb*) Avicaya (Table 1), and collectively account for 86% of the total population of the communities involved in the study (3681; Census 2012). Household density ranged from 1 – 10 people/household (average 3.5).

The exposure component also accounts for households with sensitive groups (infants <5 years, children 5-14 years, the elderly, people with disabilities and single parents) by vi7. Figure S3a shows that 32.4% of the surveyed population are students (i.e., including children 5-14 years), whilst the elderly account for 2.8% and infants 7.2%. Accordingly, sensitive groups were present in many households; children 5-14 years were present in 15% of households, infants <5 years and/or elderly people in 35%, and infants <5 years and/or elderly people as well as a single parent in 25% of households. These groups are considered sensitive because, for example, they include young children whose vulnerability relates to their general lack of knowledge and life experience for coping with water scarcity and contamination (Flanagan et al. 2011).

Also contributing to Vc2 is vi9 – vi14 (Table S1). For vi9, higher scores are obtained for households having to travel farther distances to water sources. In addition to 43% of surveyed households travelling between 0.1 km and 3 km to collect water throughout the year, due to general water scarcity in the dry season, many people have to travel even farther for water at this time. Households obtained higher vi10 and vi11 scores if they are within 0.5 km of a river assessed as CWQHR >6 and/or mine tailings/dam as they are more likely to be exposed to contamination than communities farther away. This included communities located within 0.5 km of the Antequera River (*ca*, *cb* and *ch*, Fig. 1) and the Pazña River (*ck* and *cj*).

Area of cultivated land (vi12) and number of livestock (vi13-14) are indicators that reflect information on the mainstay of many local livelihoods; 64% of surveyed households undertake some type of subsistence agriculture, with a corresponding total 160 ha of land being cultivated and thus potentially exposed to poor water quality or scarcity problems (e.g., lack of irrigation water). Around half of the surveyed households have livestock (sheep, cows, llamas, pigs, and poultry), and as mentioned previously, respondents consider that there is both insufficient water for their livestock and that poor quality water is a significant contributor to sickness and/or death of livestock.

**S1.3. Political and institutional vulnerability (Vc3; vi15 – vi18)**

The Vc3 was compiled using information on questions pertaining to levels of community organisation (vi15), the occurrence of water conflicts (vi16), relationships with and between organisations and institutions (vi17), and communities influence on water related decision-making (vi18) (Table S1). Table 1 shows that the Vc3 score for the study area was 8.05 (community score range 7.16 - 8.86), which as mentioned previously is the highest scoring Vc. However, the relative importance of Vc3 to other Vcs means that when weighted the study area Vc3 score (1.16) is the third largest contributor to Vt (Fig. 3a). Communities with the highest Vc3 scores were *cj*, *ci*, and *cc* (Fig. 1). Of particular concern is *ci*, Pazña, as it is the highest population community within the study area (1407 population, 72 people surveyed) and the high Vc3 score is primarily due to surveyed households considering they have no influence on water related decision-making, that they have no relationship with institutions and organisations (e.g., mining companies), and because they consider their community to be poorly organised.

In fact, 67% of surveyed households in the study area score highly for vi17 as they also consider that their communities do not have any relationship with mining operators in the area, nor their municipality, which might otherwise facilitate discussion of issues related to water, the environment, basic sanitation, and education. Furthermore, more than half of surveyed households (57%) are located in communities that have been involved in conflicts over water (vi16), both with other communities to get clean water and/or against mining companies or cooperatives over contamination concerns.

Although communities most directly affected by mining contamination due to their proximity to poor quality rivers (see Section 5) are often part of the *Coordinadora en defensa de la cuenca del Río Desaguadero, los lagos Uru Uru y Poopó* (CORIDUP 2015; who seek to defend and stop anthropogenic contamination of the environment that affects communities, their livelihood and future possibilities of socioeconomic development), they are not autonomous in decision-making processes regarding water and therefore obtain high vi18 scores. An example includes the passing of the Law of Mining and Metallurgy (No. 535) in May 2014, which was in fact rejected by the communities and CORIDUP.

**S1.4. Educational and cultural vulnerability (Vc4; vi19 – vi21)**

The Vc4 score for the entire study area was 5.45 (Table 1). Its weighted contribution to Vt is shown in Fig. 3a (0.64). The range of Vc4 scores for communities was large; 2.50 – 10.0, with both the highest and the lowest scores being from single households (*cf* and *cd*, respectively).

This component was based on levels of illiteracy (vi19), awareness of water risk (vi20) and training on water issues (vi21) in households (Table S1 and S2). Although nearly 90% of the surveyed population have some level of education, this is mainly at elementary level, and the remaining (mostly women with an average age of 59) have no education and are often illiterate (vi19). The potential risks of consuming poor quality water (vi20) were unknown by 19% of surveyed residents, and few people (14%) reported to have received training on water issues (vi21).

The importance of this component can be realised by considering the relationship between education and income/poverty (Flanagan et al. 2011). The overall low level of education and training reported is restricting for the regions capacity to reduce poverty and vulnerability to water issues. Generally, people with higher levels of education have better possibilities of employment and hence opportunities for higher and/or more guaranteed incomes that can positively affect their community. They might also be better informed and trained to cope with water shortages and contamination (Cutter et al. 2003; Flanagan et al. 2011).

**S1.5. Social vulnerability (Vc5; vi22 – vi25)**

The social component, Vc5, was determined on the basis of levels of migration (vi22), involvement in and importance of meetings (vi23 - vi25) (Fig. 2, Table S1). Table 1 shows that the Vc5 score for the study area was 4.60 (community score range 2.50 – 5.29), and the weighted contribution to Vt was 0.48 (Fig. 3a). This was the lowest of all Vcs and least contributor to the study area Vt, which suggests that despite weaknesses (below) the social component is the overall least vulnerable aspect of water scarcity and quality issues in the study area. The communities with the highest Vc5 score, however, and hence the most vulnerable with respect to the social component were *cb* and *cj* (Fig. 1).

An important aspect to the general social character of the study area is migration (vi22): 146 people from the participant households (250 people remaining) had left the area either permanently or temporarily (i.e., 36% of total resident and migrant population). Communities with the highest migration were *ch* and *cg*, and surveyed households within these communities obtained high vi22 scores.

Also, social vulnerability is influenced by the fact that people in the study area have lost the former typical social organisation of agrarian unions or *ayllus*, who traditionally had regular monthly meetings to discuss important issues related to their territory, production, education, health, etc. After the promulgation of the law of ‘Popular Participation’ in 1994, which resulted in the national budget being decentralised to the municipalities, peasant and indigenous groups in rural areas were instead organised in *Organizaciones Territoriales de Base* (OTBs) to enable them to have a role in the planning and use of local resources (Chaplin 2010). However, OTBs have more sporadic meetings with people being less cohesive, and they have become more a component of the political instrument as opposed to a group that defends community interests. Despite this, the majority of surveyed households (81%) attend OTB meetings (vi23), and although they were originally only attended by men, over the last 10 – 15 years women have started to attend (vi24); 72% of the surveyed population stated that women participate, although this does not necessarily mean they are acknowledged.

As part of an OTB, a community can develop projects and ask for local resources for their community from the municipality. It is therefore important that communities discuss water related issues (vi25). Water issues are considered to be a very important aspect of meetings by 58% of respondents, whereas 38% do not consider it as an important issue for discussion. As a water stressed area due to climate in particular, social organisations that do not consider water as part of their agenda effectively contribute to increasing community vulnerability as they are more unlikely to become involved in water related projects or discussion.

**S1.6. Economic vulnerability (Vc6; vi26 – vi28)**

The economic component, Vc6, is based on scoring economic activity (vi26), diversification of production (vi27) and poverty level (vi28) (Fig. 2, Table S1). The Vc6 score for the entire study area was 6.92 (community range 5.54 – 7.83; Table 1), which when weighted to Vt contributed 0.63 (Fig. 3a). The communities with the highest Vc6 scores were *ce* (7.83) and *ci* (7.35). As for Vc3, of particular concern is *ci*, Pazña, as it is the highest population community within the study area, whereby the high Vc6 score indicates the economic vulnerability of a large proportion of the surveyed population (72/250 people). Reasons for high Vc6 scores are as follows.

As mentioned in Section 2.2, the Census (2012) showed that 46.1% of the labour force in the municipality of Pazña is employed in the agriculture, livestock and fisheries sectors. Within the study area itself, agriculture and/or livestock are the largest occupational sectors, but we find that these sectors account for a maximum of 25.3% of the surveyed working labour force (Fig. S3b). This sector still represents the collective main economic activity of the surveyed work force of 144 people employing 12.5% of men and 11.8% of women. However, men in fact primarily work in technical services (i.e., automotive, plumbing, painting, etc.; 14.6% of total labour force.), and women are primarily housewives (22.9% of total surveyed labour force). The dominance of agricultural workers and housewives in relation to other sectors (Fig. S3b), especially the lack of professional sector employment (1.4% of labour force), results in higher vi26 scores for those households.

As mentioned previously, 64% of surveyed households cultivate crops (e.g., potatoes, oca, wheat, corn, and beans), which are mainly used for subsistence and/or fodder for their cattle. However, 6.5% of households only cultivate cash crops, and 13% cultivate commercial crops along with subsistence crops and/or fodder. Households that cultivate cash crops obtained a low vi27 score, for example, due to the income generated by high value quinoa (Bolivian annual Producer Price of quinoa 1373.3 US $/Ton; FAO UN 2012). On the other hand, households with low incomes (i.e., classified as poor in the 2012 Census, e.g., 59.7% of the entire population of Pazña town, *ci*) obtain high vi28 scores as they are considered more vulnerable to water issues because they are, for example, unlikely to have enough money to cover health assistance in the event of illness, or insurance if they lose their crops or animals to water related situations (Cutter et al. 2003; Flanagan et al. 2011).

**REFERENCES**

Census (2012) Instituto Nacional de Estadística, Censo de Población y Vivienda. http://datos.ine.gob.bo/binbol/RpWebEngine.exe/Portal?&BASE=CPV2012COM. Accessed 4 May 2015.

Chaplin A (2010) Social movements in Bolivia: from strength to power. Republished from Community Development Journal (2010), 45, 346 - 355. http://boliviarising.blogspot.com/2013/06/social-movements-in-bolivia-from.html. Accessed 16 April 2015.

Coordinadora en defensa de la cuenca del Río Desaguadero, los lagos Uru Uru y Poopó (CORIDUP) (2015) http://coridup.blogspot.com/. Accessed 21 April 2015.

Cutter SL (1996) Vulnerability to environmental hazards. Prog Human Geog 20: 529–39.

Flanagan B, Gregory E, Hallisey E, Heitgerd J, Lewis B (2011) A social vulnerability index for disaster management. J Homeland Sec Emerg Manag 8: Article 3.

Food and Agriculture Organization of the United Nations (FAO UN) (1985) Water quality for agriculture. http://www.fao.org/docrep/003/T0234E/T0234E00.HTM. Accessed 4 September 2014.

Food and Agriculture Organization of the United Nations (FAO UN) Statistics Division (2012) Annual Producer Prices 2012. http://faostat3.fao.org/download/P/PP/E. Accessed 22 April 2015.

Instituto Nacional de Defensa Civil. Dirección Nacional de Prevención (INDECI) (2006) Manual básico para la estimación del riesgo. http://www.indeci.gob.pe/prev_desat/pdfs/man_bas_est_riesgo.pdf. Accessed 13 February 2014.

Servicio Nacional de Meteorologia e Hidrologia – Bolivia (SENAMHI) (2014) http://www.senamhi.gob.bo/. Accessed 8 September 2014.

World Health Organization (WHO) (2003) Domestic Water Quantity, Service Level and Health. WHO/SDE/WSH/03.02. [http://www.who.int/water_sanitation_health/diseases/WSH03.02.pdf. Accessed 26 April 2014](http://www.who.int/water_sanitation_health/diseases/WSH03.02.pdf.%20Accessed%2026%20April%202014).

World Health Organization (WHO) (2011) Guidelines for Drinking-water Quality. 4^th^ Edition. ISBN 978 92 4 154815.1. <http://www.who.int/water_sanitation_health/publications/2011/dwq_guidelines>. Accessed 4 September 2014.

**Supplementary Figures**

Figure S1. Precipitation and temperatures in Poopó Town January 2013 – July 2014 (SENAMHI 2014).

Figure S2. Water source supplied to surveyed households (% of total of 72) in the study area.

Figure S3. Graphs showing a) demographics of surveyed residents (250 people), b) principle occupation of surveyed male and female work force in the study area (as % of 144 people).

**Table S1.** Scoring description and information for vulnerability components and indicators of household vulnerability assessment towards water quality and scarcity.

| Vulnerability component (Vc) and vulnerability indicators (vi) | | | *wf** | *Vc wf / 153* | *Information/data source* | Vulnerability scoring and description | | | |
| --- | --- | --- | --- | --- | --- | --- | --- | --- | --- |
|  |  |  |  |  |  | 1: Low | 2: Medium | 3: High | 4: Very high |
| Vc1: Environmental | vi1 | Main water source for human consumption | *10* |  | *Survey* | Public piped network only | Communal standpipe | Shallow well only or in combination with other sources | River and/or spring water (not controlled by any responsible party) or direct no access |
|  | vi2 | Main water source used for irrigation | *8* |  | *Survey, interviews* | Watershed source assessed as CWQHR <6 or micro-irrigation | Shallow well | Rain-fed production in wet season, or do not cultivate | River or lake assessed as CWQHR >5 |
|  | vi3 | Main water source used for livestock | *8* |  | *Survey* | Watershed source assessed as CWQHR <6, or no livestock | Shallow well | *Vigiña* | River or lake assessed as CWQHR >5, and with other sources |
|  | vi4 | Water usage per capita per day | *10* |  | *Survey* | >50 L/p/d | 21 - 50 L/p/d | 6 - 20 L/p/d | ≤5 L/p/d |
|  | vi5 | Perceived effect of water on health | *5* | *:41* | *Survey* | No illness |  |  | Illness in humans and/or animals |
| Vc2: Exposure | vi6 | Resident community population | *3* |  | *Census 2012* | <25th percentile (<15 residents) | Between 25-50th percentile (15 - 41 residents) | Between 50-75th percentile (16 - 168 residents) | >75th percentile (>168 residents) |
|  | vi7 | Presence of sensitive groups in household | *7* |  | *Survey* | No sensitive groups | Presence of people < 14 years and > 60 years | Presence of people < 5 years and > 60 years | People < 5 years, > 60 years, with disabilities, who are single head of household |
|  | vi8 | Number of people per household | *3* |  | *Survey* | <25th percentile (1 person/household) | 25th percentile (2 people/household) | 50th percentile and <75th (3 or 4 people/household) | 75th percentile or greater (5 or more people/household) |
|  | vi9 | Distance to human water source | *6* |  | *Survey* | At house | From 10 - 100 m from house | Between 101 and 999 m | 1000 m or more |
|  | vi10 | Distance to a river assessed as CWQHR 8 or more | *7* |  | *Maps and water quality assessment* | >2000 m | 1001 - 2000 m | 501-1000 m | <500 m |
|  | vi11 | Distance to mine audit/tailings/dam | *7* |  | *Maps* | >2000 m | 1001 - 2000 m | 501-1000 m | <500 m |
|  | vi12 | Cultivated area/household | *3* |  | *Survey* | <25th percentile (<1 hectare) | 25-50th percentile (1-2 hectares) | >50th up to 75th percentile (>2 up to 5 hectares) | >75th percentile (>5 hectares) |
|  | vi13 | Number of sheep/household | *3* |  | *Survey* | <25th percentile (<13 sheep) | Between 25-50th percentile (13 - 28 sheep) | >50th up to 75th percentile (>28 up to 50 sheep) | >75th percentile (>50 sheep) |
|  | vi14 | Number of cattle/household | *3* | *:42* | *Survey* | <25th percentile (<4 cattle) | Between 25-50th percentile (4 - 7 cattle) | >50th up to 75th percentile (>7 up to 10 cattle) | >75th percentile (>10 cattle) |
| Vc3: Political and Institutional | vi15 | Organisation level of community | *5* |  | *Survey, interviews* | Very organised | Fairly well organised | Poorly organised | Not organised |
|  | vi16 | Water conflicts (community) | *5* |  | *Survey, interviews* | None recently or ever | Minimal water conflicts | Some water conflicts | Many water conflicts |
|  | vi17 | Relationship between organisations and institutions | *5* |  | *Survey, interviews* | There is a definite relationship | There is some kind of relationship | Irrelevant relationship | No relationship |
|  | vi18 | Communities influence on water related decision-making | *7* | *:22* | *Survey, interviews* | High level of influence | Medium level of influence | Poor level of influence | No influence |
| Vc4: Educational and Cultural | vi19 | Illiteracy (household) | *7* |  | *Survey* | No-one in household is illiterate | Up to 12.5% of household members are illiterate | 12.5% - 25% of household members are illiterate | >25% of household members are illiterate |
|  | vi20 | Awareness of water risk (household) | *6* |  | *Survey, interviews* | Household is aware |  |  | Lack of awareness |
|  | vi21 | Training on water issues (household) | *5* | *:18* | *Survey* | 50% or more of surveyed households in the community have a family member who has training | >20% but <50% of surveyed households in the community have a family member who has training | Between 5 - 20% of surveyed households have a family member who has training | <5% of surveyed households have a family member who has received training |
| Vc5: Social | vi22 | Migration level (community) | *2* |  | *Survey* | <25th percentile (<18% of original total survey population) | Between 25-50th percentile (18-33% of original total survey population) | Between 50-75th percentile (33-48% of original total survey population) | >75th percentile (>48% of original total survey population) |
|  | vi23 | Involvement in meetings (head of household) | *4* |  | *Survey, interviews* | Head of household participates in meetings |  |  | Head of household does not participate in meetings |
|  | vi24 | Women’s participation in meetings | *5* |  | *Survey* | Women participate significantly in meetings | Women participate to a degree in meetings | Women participate very little in meetings | Women do not participate in meetings, or did not respond |
|  | vi25 | Importance of water issues at meetings | *5* | *:16* | *Survey, interviews* | Considered very important | Considered relatively important | Do not think it so important | Not addressed at meetings, or household  have no opinion |
| Vc6: Economic | vi26 | Main economic activity (household) | *8* |  | *Survey, interviews* | Professional sector (e.g., Health, Education etc.) | Mining sector, commerce or technical positions | Agricultural and farming | Elderly people, housewives or unemployed |
|  | vi27 | Diversification of production (household) | *3* |  | *Survey* | High productivity and market of products | Crops for consumption and market (25-50%) | Crops for consumption and market (up to 25%) | Crops for self-consumption, or no crops |
|  | vi28 | Poverty level including access to basic services (municipality) | *3* | *:14* | *Census 2012* | Not ranked in poverty level | Population with low poverty level rating | Population with medium poverty level rating | Population with extreme poverty level rating |

*wf: weighting factor on scale 1 (least) - 10 (highest) of relative importance of vulnerability indicators to each other.

**Table S2.** Household survey results for vulnerability assessment of water quality and scarcity (com: community; hh: household), showing hh data for vulnerability indicators (vi), vulnerability components (Vc) and total vulnerability (Vt).

| Vulnerability components (Vc) and indicators (vi) | | wf* | Community (com) code: | *ca* | | | | | | | | | | | | | *cb* | | | | | | | | | *cc* | |
| --- | --- | --- | --- | --- | --- | --- | --- | --- | --- | --- | --- | --- | --- | --- | --- | --- | --- | --- | --- | --- | --- | --- | --- | --- | --- | --- | --- |
|  |  |  | Household (hh) code: | *h1* | *h2* | *h3* | *h4* | *h5* | *h6* | *h7* | *h8* | *h9* | *h10* | *h11* | *h12* | *Mean h1-h12* | *h13* | *h14* | *h15* | *h16* | *h17* | *h18* | *h19* | *h20* | *Mean h13-h20* | *h21* | *h22* |
|  |  |  | # people/hh: | 7 | 8 | 5 | 1 | 4 | 3 | 7 | 4 | 4 | 5 | 4 | 1 | 4.4 | 5 | 4 | 6 | 3 | 5 | 7 | 1 | 2 | 4.1 | 2 | 4 |
| Vc1 | vi1 | 10 | Water source human | 3 | 3 | 1 | 1 | 3 | 3 | 1 | 1 | 1 | 3 | 1 | 4 | 2.08 | 1 | 2 | 2 | 2 | 2 | 2 | 1 | 1 | 1.63 | 1 | 1 |
|  | vi2 | 8 | Water source irrigation | 4 | 3 | 3 | 3 | 1 | 3 | 3 | 3 | 3 | 3 | 3 | 3 | 2.92 | 3 | 3 | 3 | 3 | 3 | 3 | 3 | 3 | 3.00 | 3 | 3 |
|  | vi3 | 8 | Water source livestock | 4 | 2 | 1 | 1 | 2 | 1 | 1 | 1 | 1 | 1 | 1 | 1 | 1.42 | 1 | 1 | 1 | 1 | 2 | 1 | 2 | 4 | 1.63 | 3 | 1 |
|  | vi4 | 10 | Water usage/c/d | 3 | 4 | 4 | 4 | 2 | 3 | 4 | 4 | 4 | 3 | 4 | 4 | 3.58 | 4 | 4 | 2 | 3 | 3 | 4 | 2 | 3 | 3.13 | 2 | 3 |
|  | vi5 | 5 | Effect of water on health | 4 | 1 | 4 | 1 | 4 | 4 | 4 | 1 | 4 | 1 | 1 | 4 | 2.75 | 1 | 1 | 1 | 1 | 1 | 4 | 1 | 4 | 1.75 | 4 | 1 |
|  | Vc1^ | 41 | ENVIRONMENTAL | 8.78 | 7.01 | 6.22 | 5.30 | 5.73 | 6.83 | 6.22 | 5.30 | 6.22 | 5.91 | 5.30 | 8.05 | 6.41 | 5.30 | 5.91 | 4.70 | 5.30 | 5.79 | 6.83 | 4.57 | 7.07 | 5.69 | 5.98 | 4.70 |
| Vc2 | vi6 | 3 | Resident population (com) | 4 | 4 | 4 | 4 | 4 | 4 | 4 | 4 | 4 | 4 | 4 | 4 | 4.00 | 4 | 4 | 4 | 4 | 4 | 4 | 4 | 4 | 4.00 | 2 | 2 |
|  | vi7 | 7 | Sensitive groups/hh | 2 | 3 | 3 | 4 | 3 | 3 | 2 | 3 | 2 | 2 | 1 | 1 | 2.42 | 3 | 2 | 2 | 3 | 2 | 3 | 3 | 4 | 2.75 | 1 | 3 |
|  | vi8 | 3 | Number of people/hh | 4 | 4 | 4 | 1 | 3 | 3 | 4 | 3 | 3 | 4 | 3 | 1 | 3.08 | 4 | 3 | 4 | 3 | 4 | 4 | 1 | 2 | 3.13 | 2 | 3 |
|  | vi9 | 6 | Distance: human water | 1 | 3 | 3 | 1 | 3 | 3 | 1 | 1 | 1 | 4 | 2 | 1 | 2.00 | 1 | 1 | 3 | 2 | 1 | 1 | 1 | 1 | 1.38 | 3 | 2 |
|  | vi10 | 7 | Distance: river CWQHR8+ | 4 | 4 | 4 | 4 | 4 | 4 | 4 | 4 | 4 | 4 | 4 | 4 | 4.00 | 4 | 4 | 4 | 4 | 4 | 4 | 4 | 4 | 4.00 | 2 | 2 |
|  | vi11 | 7 | Distance: mine tails etc. | 4 | 4 | 4 | 4 | 4 | 4 | 4 | 4 | 4 | 4 | 4 | 4 | 4.00 | 4 | 4 | 4 | 4 | 4 | 4 | 4 | 4 | 4.00 | 2 | 2 |
|  | vi12 | 3 | Cultivated area/hh | 1 | 1 | 1 | 1 | 1 | 1 | 1 | 1 | 1 | 1 | 1 | 1 | 1.00 | 2 | 2 | 1 | 1 | 2 | 3 | 4 | 2 | 2.13 | 3 | 4 |
|  | vi13 | 3 | Sheep/hh | 4 | 1 | 1 | 1 | 2 | 1 | 1 | 1 | 1 | 1 | 1 | 1 | 1.33 | 1 | 3 | 1 | 1 | 1 | 1 | 1 | 2 | 1.38 | 4 | 1 |
|  | vi14 | 3 | Cattle/hh | 1 | 1 | 1 | 1 | 4 | 1 | 1 | 1 | 1 | 1 | 1 | 1 | 1.25 | 1 | 3 | 1 | 1 | 2 | 1 | 1 | 1 | 1.38 | 2 | 1 |
|  | Vc2 | 42 | EXPOSURE | 7.02 | 7.62 | 7.62 | 6.79 | 8.15 | 7.44 | 6.49 | 6.73 | 6.31 | 7.56 | 6.25 | 5.54 | 6.96 | 7.08 | 7.20 | 7.20 | 7.08 | 6.85 | 7.26 | 6.90 | 7.32 | 7.11 | 5.48 | 5.60 |
| Vc3 | vi15 | 5 | Organisation level (com) | 2 | 2 | 2 | 2 | 2 | 2 | 2 | 2 | 2 | 2 | 2 | 2 | 2.00 | 2 | 2 | 2 | 2 | 2 | 2 | 2 | 2 | 2.00 | 2 | 2 |
|  | vi16 | 5 | Water conflicts (com) | 1 | 2 | 4 | 4 | 4 | 1 | 4 | 1 | 4 | 2 | 1 | 4 | 2.67 | 4 | 4 | 4 | 1 | 2 | 4 | 1 | 4 | 3.00 | 4 | 1 |
|  | vi17 | 5 | Institution organisation (com) | 4 | 2 | 2 | 2 | 4 | 4 | 2 | 4 | 4 | 2 | 2 | 4 | 3.00 | 2 | 2 | 3 | 4 | 4 | 4 | 2 | 2 | 2.88 | 4 | 4 |
|  | vi18 | 7 | Water related decisions (com) | 4 | 4 | 4 | 4 | 4 | 4 | 4 | 4 | 4 | 4 | 4 | 4 | 4.00 | 4 | 4 | 4 | 4 | 4 | 4 | 4 | 4 | 4.00 | 4 | 4 |
|  | Vc3 | 22 | POLITICAL & INSTITUTIONAL | 7.16 | 6.59 | 7.73 | 7.73 | 8.86 | 7.16 | 7.73 | 7.16 | 8.86 | 6.59 | 6.02 | 8.86 | 7.54 | 7.73 | 7.73 | 8.30 | 7.16 | 7.73 | 8.86 | 6.02 | 7.73 | 7.66 | 8.86 | 7.16 |
| Vc4 | vi19 | 7 | Illiteracy | 1 | 1 | 1 | 4 | 1 | 1 | 3 | 1 | 3 | 1 | 1 | 1 | 1.58 | 1 | 3 | 1 | 1 | 3 | 1 | 1 | 1 | 1.50 | 1 | 3 |
|  | vi20 | 6 | Water risk aware | 1 | 1 | 1 | 4 | 1 | 1 | 1 | 1 | 1 | 4 | 1 | 1 | 1.50 | 4 | 1 | 1 | 4 | 1 | 4 | 1 | 1 | 2.13 | 1 | 4 |
|  | vi21 | 5 | Training | 3 | 3 | 3 | 3 | 3 | 3 | 3 | 3 | 3 | 3 | 3 | 3 | 3.00 | 4 | 4 | 4 | 4 | 4 | 4 | 4 | 4 | 4.00 | 2 | 2 |
|  | Vc4 | 18 | EDUCATIONAL & CULTURAL | 3.89 | 3.89 | 3.89 | 9.31 | 3.89 | 3.89 | 5.83 | 3.89 | 5.83 | 6.39 | 3.89 | 3.89 | 4.87 | 7.08 | 6.53 | 4.58 | 7.08 | 6.53 | 7.08 | 4.58 | 4.58 | 6.01 | 3.19 | 7.64 |
| Vc5 | vi22 | 2 | Migration (com) | 1 | 1 | 1 | 1 | 1 | 1 | 1 | 1 | 1 | 1 | 1 | 1 | 1.00 | 2 | 2 | 2 | 2 | 2 | 2 | 2 | 2 | 2.00 | 4 | 4 |
|  | vi23 | 4 | Involvement in meetings | 1 | 4 | 1 | 1 | 1 | 1 | 1 | 1 | 1 | 1 | 1 | 1 | 1.25 | 1 | 1 | 1 | 1 | 1 | 1 | 1 | 4 | 1.38 | 1 | 1 |
|  | vi24 | 5 | Women in meetings | 1 | 4 | 3 | 1 | 1 | 1 | 4 | 1 | 3 | 1 | 1 | 1 | 1.83 | 4 | 4 | 1 | 1 | 1 | 4 | 2 | 4 | 2.63 | 1 | 1 |
|  | vi25 | 5 | Water issues at meetings | 3 | 4 | 1 | 1 | 3 | 3 | 4 | 1 | 1 | 3 | 1 | 1 | 2.17 | 3 | 3 | 4 | 1 | 1 | 1 | 4 | 1 | 2.25 | 1 | 1 |
|  | Vc5 | 16 | SOCIAL | 4.06 | 9.06 | 4.06 | 2.50 | 4.06 | 4.06 | 7.19 | 2.50 | 4.06 | 4.06 | 2.50 | 2.50 | 4.22 | 6.72 | 6.72 | 5.16 | 2.81 | 2.81 | 5.16 | 5.94 | 7.03 | 5.29 | 3.44 | 3.44 |
| Vc6 | vi26 | 8 | Main economic activity | 2 | 2 | 2 | 2 | 3 | 2 | 2 | 2 | 2 | 2 | 2 | 1 | 2.00 | 2 | 2 | 2 | 2 | 3 | 2 | 4 | 2 | 2.38 | 3 | 3 |
|  | vi27 | 3 | Diversification production | 3 | 4 | 4 | 4 | 3 | 4 | 4 | 4 | 4 | 4 | 4 | 4 | 3.83 | 4 | 4 | 4 | 4 | 2 | 2 | 2 | 2 | 3.00 | 2 | 2 |
|  | vi28 | 3 | Poverty level (com) | 3 | 3 | 3 | 3 | 3 | 3 | 3 | 3 | 3 | 3 | 3 | 3 | 3.00 | 3 | 3 | 3 | 3 | 3 | 3 | 3 | 3 | 3.00 | 3 | 3 |
|  | Vc6 | 12 | ECONOMIC | 6.07 | 6.61 | 6.61 | 6.61 | 7.50 | 6.61 | 6.61 | 6.61 | 6.61 | 6.61 | 6.61 | 5.18 | 6.52 | 6.61 | 6.61 | 6.61 | 6.61 | 6.96 | 5.54 | 8.39 | 5.54 | 6.61 | 6.96 | 6.96 |
| Vt^^ | | 153 | TOTAL VULNERABILITY | 6.75 | 6.93 | 6.36 | 6.36 | 6.62 | 6.39 | 6.60 | 5.62 | 6.39 | 6.39 | 5.33 | 6.14 | 6.32 | 6.62 | 6.75 | 6.11 | 6.13 | 6.24 | 6.98 | 5.92 | 6.80 | 6.44 | 5.75 | 5.72 |

* wf: weighting score on scale 1 (least) - 10 (highest) of relative importance of vulnerability indicators to each other.

^ Example: Vc1 h1 = ([vi1 h1/4 × vi1 wf/Vc1 wf] +...[vi5 h1/4 × vi5 wf/Vc1 wf]) × 10

^^ Example Vt h1 = ([vi1 h1/4 × vi1 wf/Vt wf] +...[vi28 h1/4 × vi28 wf/Vt wf]) × 10

| Com. code: | | *cc* | | | *cd* | *ce* | | | | | | | *cf* | *cg* | | | | | *ch* | | | | *ci* | | | | | | | | |
| --- | --- | --- | --- | --- | --- | --- | --- | --- | --- | --- | --- | --- | --- | --- | --- | --- | --- | --- | --- | --- | --- | --- | --- | --- | --- | --- | --- | --- | --- | --- | --- |
| hh code: | | *h23* | *h24* | *Mean h21-h24* | *h25* | *h26* | *h27* | *h28* | *h29* | *h30* | *h31* | *Mean h26-h31* | *h32* | *h33* | *h34* | *h35* | *h36* | *Mean h33-h36* | *h37* | *h38* | *h39* | *Mean h37-h39* | *h40* | *h41* | *h42* | *h43* | *h44* | *h45* | *h46* | *h47* | *h48* |
| # people/hh: | | 4 | 1 | 2.8 | 20 | 1 | 2 | 2 | 6 | 4 | 1 | 2.7 | 3 | 1 | 2 | 2 | 5 | 2.5 | 2 | 3 | 2 | 2.3 | 3 | 2 | 3 | 4 | 1 | 2 | 3 | 6 | 2 |
| Vc1 | vi1 | 1 | 4 | 1.75 | 3 | 4 | 4 | 3 | 1 | 3 | 4 | 3.17 | 3 | 1 | 1 | 3 | 1 | 1.50 | 4 | 1 | 1 | 2.00 | 3 | 1 | 1 | 3 | 3 | 3 | 1 | 1 | 1 |
|  | vi2 | 3 | 3 | 3.00 | 4 | 3 | 3 | 3 | 3 | 3 | 3 | 3.00 | 4 | 3 | 1 | 3 | 4 | 2.75 | 3 | 3 | 3 | 3.00 | 3 | 3 | 3 | 4 | 3 | 3 | 3 | 3 | 3 |
|  | vi3 | 1 | 2 | 1.75 | 1 | 1 | 1 | 1 | 1 | 1 | 1 | 1.00 | 3 | 1 | 1 | 4 | 1 | 1.75 | 4 | 4 | 4 | 4.00 | 4 | 1 | 4 | 4 | 1 | 1 | 1 | 1 | 1 |
|  | vi4 | 3 | 2 | 2.50 | 4 | 4 | 4 | 3 | 4 | 4 | 3 | 3.67 | 4 | 3 | 4 | 3 | 2 | 3.00 | 3 | 3 | 4 | 3.33 | 3 | 2 | 3 | 3 | 3 | 3 | 3 | 3 | 3 |
|  | vi5 | 1 | 1 | 1.75 | 1 | 4 | 1 | 1 | 4 | 1 | 4 | 2.50 | 4 | 1 | 4 | 1 | 4 | 2.50 | 4 | 1 | 4 | 3.00 | 4 | 4 | 4 | 4 | 1 | 4 | 1 | 4 | 1 |
|  | Vc1 | 4.70 | 6.40 | 5.44 | 7.01 | 8.05 | 7.13 | 5.91 | 6.22 | 6.52 | 7.44 | 6.88 | 8.90 | 4.70 | 5.24 | 7.38 | 5.49 | 5.70 | 8.90 | 6.16 | 7.68 | 7.58 | 8.29 | 5.00 | 7.07 | 8.78 | 5.91 | 6.83 | 4.70 | 5.61 | 4.70 |
| Vc2 | vi6 | 2 | 2 | 2.00 | 2 | 3 | 3 | 3 | 3 | 3 | 3 | 3.00 | 2 | 3 | 3 | 3 | 3 | 3.00 | 1 | 1 | 1 | 1.00 | 4 | 4 | 4 | 4 | 4 | 4 | 4 | 4 | 4 |
|  | vi7 | 3 | 4 | 2.75 | 1 | 1 | 3 | 1 | 3 | 3 | 1 | 2.00 | 3 | 4 | 1 | 3 | 2 | 2.50 | 3 | 3 | 4 | 3.33 | 1 | 4 | 2 | 3 | 4 | 4 | 4 | 3 | 1 |
|  | vi8 | 3 | 1 | 2.25 | 2 | 1 | 2 | 2 | 4 | 3 | 1 | 2.17 | 3 | 1 | 2 | 2 | 4 | 2.25 | 2 | 3 | 2 | 2.33 | 3 | 2 | 3 | 3 | 1 | 2 | 3 | 4 | 2 |
|  | vi9 | 1 | 1 | 1.75 | 3 | 1 | 1 | 1 | 2 | 2 | 2 | 1.50 | 2 | 3 | 4 | 3 | 3 | 3.25 | 4 | 1 | 1 | 2.00 | 1 | 1 | 1 | 1 | 1 | 1 | 1 | 1 | 2 |
|  | vi10 | 2 | 2 | 2.00 | 1 | 1 | 1 | 1 | 1 | 1 | 1 | 1.00 | 2 | 1 | 1 | 1 | 1 | 1.00 | 3 | 3 | 3 | 3.00 | 3 | 3 | 3 | 3 | 3 | 3 | 3 | 3 | 3 |
|  | vi11 | 2 | 2 | 2.00 | 1 | 1 | 1 | 1 | 1 | 1 | 1 | 1.00 | 1 | 1 | 1 | 1 | 1 | 1.00 | 1 | 1 | 1 | 1.00 | 1 | 1 | 1 | 1 | 1 | 1 | 1 | 1 | 1 |
|  | vi12 | 1 | 1 | 2.25 | 1 | 1 | 2 | 3 | 1 | 1 | 1 | 1.50 | 2 | 3 | 3 | 1 | 2 | 2.25 | 4 | 2 | 1 | 2.33 | 1 | 1 | 3 | 3 | 4 | 4 | 1 | 2 | 1 |
|  | vi13 | 4 | 1 | 2.50 | 1 | 2 | 3 | 3 | 4 | 1 | 1 | 2.33 | 1 | 1 | 2 | 2 | 4 | 2.25 | 1 | 1 | 1 | 1.00 | 1 | 1 | 1 | 3 | 1 | 1 | 1 | 1 | 1 |
|  | vi14 | 1 | 1 | 1.25 | 1 | 1 | 2 | 1 | 4 | 1 | 3 | 2.00 | 1 | 1 | 1 | 1 | 1 | 1.00 | 2 | 2 | 1 | 1.67 | 1 | 1 | 2 | 3 | 1 | 1 | 1 | 1 | 1 |
|  | Vc2 | 5.24 | 4.76 | 5.27 | 3.57 | 3.04 | 4.58 | 3.75 | 5.65 | 4.40 | 3.57 | 4.17 | 4.82 | 5.18 | 4.64 | 4.76 | 5.24 | 4.96 | 6.13 | 4.88 | 4.76 | 5.26 | 4.23 | 5.30 | 5.18 | 6.13 | 5.65 | 5.83 | 5.48 | 5.42 | 4.40 |
| Vc3 | vi15 | 2 | 2 | 2.00 | 2 | 2 | 2 | 2 | 2 | 2 | 2 | 2.00 | 2 | 2 | 2 | 2 | 2 | 2.00 | 2 | 2 | 2 | 2.00 | 3 | 3 | 3 | 3 | 3 | 3 | 3 | 3 | 3 |
|  | vi16 | 4 | 4 | 3.25 | 3 | 4 | 4 | 1 | 1 | 4 | 4 | 3.00 | 3 | 2 | 4 | 1 | 4 | 2.75 | 1 | 4 | 1 | 2.00 | 2 | 4 | 4 | 2 | 1 | 1 | 1 | 4 | 1 |
|  | vi17 | 4 | 4 | 4.00 | 2 | 4 | 4 | 4 | 3 | 4 | 3 | 3.67 | 4 | 4 | 2 | 4 | 2 | 3.00 | 4 | 4 | 4 | 4.00 | 4 | 4 | 4 | 4 | 4 | 4 | 4 | 3 | 3 |
|  | vi18 | 4 | 4 | 4.00 | 4 | 4 | 4 | 4 | 4 | 4 | 4 | 4.00 | 4 | 4 | 4 | 4 | 4 | 4.00 | 4 | 4 | 4 | 4.00 | 4 | 4 | 4 | 4 | 4 | 4 | 4 | 4 | 4 |
|  | Vc3 | 8.86 | 8.86 | 8.44 | 7.16 | 8.86 | 8.86 | 7.16 | 6.59 | 8.86 | 8.30 | 8.11 | 8.30 | 7.73 | 7.73 | 7.16 | 7.73 | 7.59 | 7.16 | 8.86 | 7.16 | 7.73 | 8.30 | 9.43 | 9.43 | 8.30 | 7.73 | 7.73 | 7.73 | 8.86 | 7.16 |
| Vc4 | vi19 | 3 | 1 | 2.00 | 1 | 1 | 1 | 4 | 1 | 4 | 4 | 2.50 | 4 | 1 | 1 | 4 | 1 | 1.75 | 4 | 1 | 1 | 2.00 | 4 | 4 | 1 | 1 | 4 | 4 | 1 | 1 | 1 |
|  | vi20 | 1 | 1 | 1.75 | 1 | 1 | 1 | 1 | 4 | 1 | 1 | 1.50 | 4 | 1 | 1 | 4 | 1 | 1.75 | 1 | 1 | 1 | 1.00 | 1 | 1 | 4 | 1 | 1 | 1 | 1 | 1 | 1 |
|  | vi21 | 2 | 2 | 2.00 | 1 | 3 | 3 | 3 | 3 | 3 | 3 | 3.00 | 4 | 1 | 1 | 1 | 1 | 1.00 | 4 | 4 | 4 | 4.00 | 4 | 4 | 4 | 4 | 4 | 4 | 4 | 4 | 4 |
|  | Vc4 | 5.14 | 3.19 | 4.79 | 2.50 | 3.89 | 3.89 | 6.81 | 6.39 | 6.81 | 6.81 | 5.76 | 10.0 | 2.50 | 2.50 | 7.92 | 2.50 | 3.85 | 7.50 | 4.58 | 4.58 | 5.56 | 7.50 | 7.50 | 7.08 | 4.58 | 7.50 | 7.50 | 4.58 | 4.58 | 4.58 |
| Vc5 | vi22 | 4 | 4 | 4.00 | 1 | 3 | 3 | 3 | 3 | 3 | 3 | 3.00 | 2 | 4 | 4 | 4 | 4 | 4.00 | 4 | 4 | 4 | 4.00 | 3 | 3 | 3 | 3 | 3 | 3 | 3 | 3 | 3 |
|  | vi23 | 1 | 1 | 1.00 | 1 | 1 | 1 | 1 | 1 | 1 | 1 | 1.00 | 1 | 1 | 1 | 1 | 1 | 1.00 | 4 | 1 | 1 | 2.00 | 1 | 1 | 4 | 1 | 1 | 1 | 1 | 4 | 1 |
|  | vi24 | 1 | 1 | 1.00 | 1 | 1 | 1 | 1 | 2 | 1 | 1 | 1.17 | 1 | 1 | 1 | 1 | 1 | 1.00 | 1 | 1 | 1 | 1.00 | 3 | 1 | 1 | 2 | 1 | 1 | 1 | 1 | 1 |
|  | vi25 | 1 | 4 | 1.75 | 1 | 1 | 1 | 4 | 1 | 1 | 1 | 1.50 | 1 | 1 | 1 | 4 | 3 | 2.25 | 1 | 1 | 4 | 2.00 | 1 | 1 | 1 | 3 | 4 | 4 | 3 | 1 | 3 |
|  | Vc5 | 3.44 | 5.78 | 4.02 | 2.50 | 3.13 | 3.13 | 5.47 | 3.91 | 3.13 | 3.13 | 3.65 | 2.81 | 3.44 | 3.44 | 5.78 | 5.00 | 4.41 | 5.31 | 3.44 | 5.78 | 4.84 | 4.69 | 3.13 | 5.00 | 5.47 | 5.47 | 5.47 | 4.69 | 5.00 | 4.69 |
| Vc | vi26 | 3 | 2 | 2.75 | 2 | 4 | 3 | 3 | 3 | 3 | 3 | 3.17 | 2 | 4 | 2 | 2 | 3 | 2.75 | 2 | 2 | 3 | 2.33 | 2 | 2 | 2 | 3 | 4 | 3 | 2 | 3 | 2 |
|  | vi27 | 2 | 4 | 2.50 | 3 | 2 | 3 | 2 | 4 | 4 | 4 | 3.17 | 3 | 2 | 3 | 2 | 3 | 2.50 | 3 | 2 | 3 | 2.67 | 4 | 4 | 2 | 2 | 2 | 2 | 4 | 2 | 4 |
|  | vi28 | 3 | 3 | 3.00 | 3 | 3 | 3 | 3 | 3 | 3 | 3 | 3.00 | 3 | 3 | 3 | 3 | 3 | 3.00 | 3 | 3 | 3 | 3.00 | 3 | 3 | 3 | 3 | 3 | 3 | 3 | 3 | 3 |
|  | Vc6 | 6.96 | 6.61 | 6.88 | 6.07 | 8.39 | 7.50 | 6.96 | 8.04 | 8.04 | 8.04 | 7.83 | 6.07 | 8.39 | 6.07 | 5.54 | 7.50 | 6.88 | 6.07 | 5.54 | 7.50 | 6.37 | 6.61 | 6.61 | 5.54 | 6.96 | 8.39 | 6.96 | 6.61 | 6.96 | 6.61 |
| Vt | | 5.57 | 5.88 | 5.73 | 5.00 | 5.82 | 5.92 | 5.65 | 6.06 | 6.09 | 6.03 | 5.93 | 6.93 | 5.21 | 5.00 | 6.36 | 5.52 | 5.52 | 7.09 | 5.67 | 6.23 | 6.33 | 6.55 | 5.96 | 6.54 | 6.98 | 6.47 | 6.63 | 5.51 | 5.96 | 5.13 |

| Com. code: | | *ci* | | | | | | | | | | | | | | | *cj* | | | *ck* | | | | | | | *cl* | *cm* | *Study area* |
| --- | --- | --- | --- | --- | --- | --- | --- | --- | --- | --- | --- | --- | --- | --- | --- | --- | --- | --- | --- | --- | --- | --- | --- | --- | --- | --- | --- | --- | --- |
| hh code: | | *h49* | *h50* | *h51* | *h52* | *h53* | *h54* | *h55* | *h56* | *h57* | *h58* | *h59* | *h60* | *h61* | *h62* | *Mean h40-h62* | *h63* | *h64* | *Mean h63-h64* | *h65* | *h66* | *h67* | *h68* | *h69* | *h70* | *Mean h65-h70* | *h71* | *h72* | *Mean h1-h72* |
| # people/hh: | | 4 | 1 | 2 | 2 | 8 | 6 | 6 | 2 | 1 | 1 | 1 | 1 | 10 | 1 | 3.1 | 3 | 3 | 3 | 8 | 7 | 5 | 1 | 2 | 8 | 5.2 | 4 | 2 | 3.5 |
| Vc1 | vi1 | 3 | 1 | 1 | 3 | 1 | 1 | 1 | 1 | 1 | 1 | 1 | 1 | 1 | 1 | 1.52 | 1 | 1 | 1.00 | 3 | 3 | 1 | 2 | 1 | 3 | 2.17 | 1 | 3 | 1.89 |
|  | vi2 | 3 | 3 | 3 | 2 | 3 | 3 | 3 | 3 | 3 | 3 | 3 | 3 | 3 | 3 | 3.00 | 3 | 3 | 3.00 | 3 | 3 | 3 | 3 | 3 | 3 | 3.00 | 3 | 3 | 3.00 |
|  | vi3 | 4 | 1 | 2 | 2 | 2 | 1 | 1 | 2 | 1 | 4 | 1 | 1 | 1 | 1 | 1.83 | 4 | 2 | 3.00 | 1 | 2 | 4 | 3 | 3 | 4 | 2.83 | 2 | 2 | 1.88 |
|  | vi4 | 4 | 3 | 4 | 2 | 4 | 4 | 4 | 2 | 2 | 2 | 4 | 3 | 4 | 4 | 3.13 | 3 | 4 | 3.50 | 4 | 3 | 3 | 3 | 3 | 4 | 3.33 | 3 | 3 | 3.26 |
|  | vi5 | 1 | 1 | 1 | 4 | 4 | 1 | 1 | 1 | 4 | 4 | 1 | 1 | 4 | 1 | 2.43 | 4 | 4 | 4.00 | 4 | 4 | 4 | 4 | 4 | 4 | 4.00 | 1 | 4 | 2.58 |
|  | Vc1 | 7.99 | 4.70 | 5.79 | 6.22 | 6.71 | 5.30 | 5.30 | 4.57 | 5.00 | 6.46 | 5.30 | 4.70 | 6.22 | 5.30 | 5.93 | 7.07 | 6.71 | 6.89 | 7.44 | 7.32 | 7.07 | 7.20 | 6.59 | 8.90 | 7.42 | 5.18 | 7.32 | 6.31 |
| Vc2 | vi6 | 4 | 4 | 4 | 4 | 4 | 4 | 4 | 4 | 4 | 4 | 4 | 4 | 4 | 4 | 4.00 | 1 | 1 | 1.00 | 2 | 2 | 2 | 2 | 2 | 2 | 2.00 | 3 | 2 | 3.28 |
|  | vi7 | 2 | 4 | 4 | 4 | 1 | 3 | 3 | 4 | 4 | 1 | 4 | 4 | 3 | 4 | 3.09 | 1 | 1 | 1.00 | 3 | 3 | 1 | 1 | 4 | 2 | 2.33 | 1 | 1 | 2.60 |
|  | vi8 | 3 | 1 | 2 | 2 | 4 | 4 | 4 | 2 | 1 | 1 | 1 | 1 | 4 | 1 | 2.35 | 3 | 3 | 3.00 | 4 | 4 | 4 | 1 | 2 | 4 | 3.17 | 3 | 2 | 2.63 |
|  | vi9 | 2 | 1 | 3 | 1 | 1 | 1 | 3 | 1 | 1 | 2 | 1 | 1 | 4 | 1 | 1.43 | 1 | 1 | 1.00 | 1 | 4 | 2 | 4 | 4 | 3 | 3.00 | 2 | 4 | 1.86 |
|  | vi10 | 3 | 3 | 3 | 3 | 3 | 3 | 3 | 3 | 3 | 3 | 3 | 3 | 3 | 3 | 3.00 | 3 | 3 | 3.00 | 4 | 4 | 4 | 4 | 4 | 4 | 4.00 | 1 | 1 | 2.93 |
|  | vi11 | 1 | 1 | 1 | 1 | 1 | 1 | 1 | 1 | 1 | 1 | 1 | 1 | 1 | 1 | 1.00 | 1 | 1 | 1.00 | 1 | 1 | 1 | 1 | 1 | 1 | 1.00 | 1 | 1 | 1.89 |
|  | vi12 | 1 | 1 | 1 | 1 | 1 | 1 | 1 | 1 | 1 | 1 | 1 | 1 | 1 | 1 | 1.48 | 4 | 1 | 2.50 | 1 | 4 | 1 | 2 | 4 | 3 | 2.50 | 1 | 4 | 1.74 |
|  | vi13 | 2 | 1 | 1 | 1 | 1 | 1 | 1 | 1 | 1 | 1 | 1 | 1 | 1 | 1 | 1.13 | 1 | 1 | 1.00 | 1 | 1 | 3 | 3 | 1 | 3 | 2.00 | 3 | 1 | 1.51 |
|  | vi14 | 2 | 1 | 1 | 1 | 2 | 1 | 1 | 1 | 1 | 1 | 1 | 1 | 1 | 1 | 1.22 | 4 | 1 | 2.50 | 1 | 1 | 1 | 4 | 1 | 2 | 1.67 | 4 | 1 | 1.42 |
|  | Vc2 | 5.36 | 5.12 | 6.01 | 5.30 | 4.58 | 5.24 | 5.95 | 5.30 | 5.12 | 4.23 | 5.12 | 5.12 | 6.31 | 5.12 | 5.28 | 4.76 | 3.69 | 4.23 | 5.30 | 6.90 | 5.18 | 6.07 | 6.96 | 6.49 | 6.15 | 4.46 | 4.46 | 5.64 |
| Vc3 | vi15 | 3 | 3 | 3 | 3 | 3 | 3 | 3 | 3 | 3 | 3 | 3 | 3 | 3 | 3 | 3.00 | 2 | 2 | 2.00 | 2 | 2 | 2 | 2 | 2 | 2 | 2.00 | 2 | 2 | 2.32 |
|  | vi16 | 4 | 4 | 1 | 4 | 1 | 4 | 1 | 4 | 4 | 4 | 4 | 2 | 4 | 4 | 2.83 | 4 | 4 | 4.00 | 4 | 1 | 2 | 1 | 4 | 1 | 2.17 | 2 | 3 | 2.79 |
|  | vi17 | 3 | 2 | 4 | 4 | 4 | 4 | 2 | 4 | 4 | 4 | 4 | 3 | 3 | 3 | 3.57 | 4 | 4 | 4.00 | 4 | 4 | 4 | 4 | 4 | 4 | 4.00 | 4 | 4 | 3.46 |
|  | vi18 | 4 | 4 | 4 | 4 | 4 | 4 | 4 | 4 | 4 | 4 | 4 | 4 | 4 | 4 | 4.00 | 4 | 4 | 4.00 | 4 | 4 | 4 | 4 | 4 | 4 | 4.00 | 4 | 4 | 4.00 |
|  | Vc3 | 8.86 | 8.30 | 7.73 | 9.43 | 7.73 | 9.43 | 6.59 | 9.43 | 9.43 | 9.43 | 9.43 | 7.73 | 8.86 | 8.86 | 8.52 | 8.86 | 8.86 | 8.86 | 8.86 | 7.16 | 7.73 | 7.16 | 8.86 | 7.16 | 7.82 | 7.73 | 8.30 | 8.05 |
| Vc4 | vi19 | 1 | 1 | 1 | 4 | 1 | 3 | 1 | 1 | 1 | 1 | 1 | 4 | 3 | 4 | 2.09 | 1 | 1 | 1.00 | 1 | 1 | 3 | 1 | 1 | 1 | 1.33 | 3 | 1 | 1.86 |
|  | vi20 | 1 | 1 | 1 | 1 | 1 | 1 | 1 | 1 | 1 | 1 | 4 | 1 | 4 | 4 | 1.52 | 1 | 1 | 1.00 | 1 | 1 | 1 | 4 | 1 | 1 | 1.50 | 1 | 1 | 1.58 |
|  | vi21 | 4 | 4 | 4 | 4 | 4 | 4 | 4 | 4 | 4 | 4 | 4 | 4 | 4 | 4 | 4.00 | 4 | 4 | 4.00 | 3 | 3 | 3 | 3 | 3 | 3 | 3.00 | 4 | 4 | 3.35 |
|  | Vc4 | 4.58 | 4.58 | 4.58 | 7.50 | 4.58 | 6.53 | 4.58 | 4.58 | 4.58 | 4.58 | 7.08 | 7.50 | 9.03 | 10.0 | 6.07 | 4.58 | 4.58 | 4.48 | 3.89 | 3.89 | 5.83 | 6.39 | 3.89 | 3.89 | 4.63 | 6.53 | 4.58 | 5.45 |
| Vc5 | vi22 | 3 | 3 | 3 | 3 | 3 | 3 | 3 | 3 | 3 | 3 | 3 | 3 | 3 | 3 | 3.00 | 3 | 3 | 3.00 | 2 | 2 | 2 | 2 | 2 | 2 | 2.00 | 2 | 1 | 2.54 |
|  | vi23 | 1 | 1 | 4 | 1 | 1 | 1 | 1 | 4 | 4 | 4 | 4 | 4 | 1 | 1 | 2.04 | 4 | 1 | 2.50 | 1 | 1 | 4 | 1 | 1 | 1 | 1.50 | 1 | 1 | 1.54 |
|  | vi24 | 1 | 1 | 1 | 1 | 1 | 1 | 1 | 1 | 4 | 1 | 4 | 2 | 1 | 3 | 1.52 | 4 | 1 | 2.50 | 1 | 2 | 3 | 1 | 1 | 1 | 1.50 | 1 | 1 | 1.58 |
|  | vi25 | 4 | 4 | 4 | 1 | 1 | 3 | 3 | 4 | 1 | 1 | 1 | 1 | 1 | 3 | 2.30 | 1 | 1 | 1.00 | 1 | 1 | 4 | 4 | 1 | 1 | 2.00 | 3 | 1 | 2.06 |
|  | Vc5 | 5.47 | 5.47 | 7.34 | 3.13 | 3.13 | 4.69 | 4.69 | 7.34 | 7.34 | 5.00 | 7.34 | 5.78 | 3.13 | 6.25 | 5.20 | 7.34 | 3.13 | 5.23 | 2.81 | 3.59 | 8.59 | 5.16 | 2.81 | 2.81 | 4.30 | 4.38 | 2.50 | 4.60 |
| Vc6 | vi26 | 2 | 4 | 2 | 4 | 3 | 2 | 2 | 4 | 4 | 2 | 2 | 4 | 2 | 4 | 2.78 | 2 | 2 | 2.00 | 2 | 2 | 3 | 3 | 3 | 3 | 2.67 | 3 | 3 | 2.57 |
|  | vi27 | 4 | 4 | 4 | 2 | 2 | 4 | 4 | 2 | 4 | 4 | 4 | 4 | 4 | 4 | 3.30 | 2 | 2 | 2.00 | 2 | 2 | 2 | 3 | 3 | 2 | 2.33 | 2 | 2 | 3.07 |
|  | vi28 | 3 | 3 | 3 | 3 | 3 | 3 | 3 | 3 | 3 | 3 | 3 | 3 | 3 | 3 | 3.00 | 3 | 3 | 3.00 | 3 | 3 | 3 | 3 | 3 | 3 | 3.00 | 3 | 3 | 3.00 |
|  | Vc6 | 6.61 | 9.46 | 6.61 | 8.39 | 6.96 | 6.61 | 6.61 | 8.39 | 9.46 | 6.61 | 6.61 | 9.46 | 6.61 | 9.46 | 7.35 | 5.54 | 5.54 | 5.54 | 5.54 | 5.54 | 6.96 | 7.50 | 7.50 | 6.96 | 6.67 | 6.96 | 6.96 | 6.92 |
| Vt | | 6.60 | 5.83 | 6.23 | 6.45 | 5.67 | 6.08 | 5.64 | 6.11 | 6.27 | 5.92 | 6.39 | 6.13 | 6.67 | 6.80 | 6.20 | 6.29 | 5.46 | 5.87 | 5.98 | 6.23 | 6.65 | 6.60 | 6.39 | 6.58 | 6.41 | 5.59 | 5.82 | 6.15 |

**Table S3.** Qualitative description of vulnerability categories for describing total vulnerability (Vt) (after INDECI, 2006).

| Level | Vt range | Description |
| --- | --- | --- |
| Low | <2.5 | Communities with i) access to basic services (such as potable water supply and sewage system), ii) the amount of water they have available for use is sufficient to meet at least their basic needs (i.e., for consumption, food preparation and hygiene), iii) general education, vi) education on water issues, v) steady income, vi) do not suffer ill health (including to livestock) due to water contamination, vii) high level of social organisation and autonomy in decision-making. |
| Medium | 2.5 – 4.9 | Communities have i) access to basic services (such as potable water supply and sewage system), ii) the amount of water they have available for use is usually sufficient to meet their basic needs, iii) at least high school education, iv) knowledge and training on water issues, v) dependable income, vi) no reported ill-health (including to livestock) due to water contamination, vii) social organisation and some level of autonomy in decision-making. |
| High | 5.0 – 7.5 | Communities have i) limited access to basic services (such as potable water supply and sewage system), ii) the amount of water they have available for use is insufficient to meet their basic needs (i.e., for consumption, food preparation and hygiene), iii) primary education, iv) no training on water issues, v) marginal income, vi) many people perceive they and/or their livestock have suffered ill-health due to water contamination, vii) some level of social organisation but are not autonomous in decision-making. |
| Very high | >7.5 | Communities have i) no access to basic services (such as water supply and sewage system), ii) the amount of water they have available for use is insufficient to meet their basic needs, iii) high illiteracy, iv) no access to training on water issues, v) a survival economy, vi) believe they have suffered ill-health due to water contamination, vii) no strong social organisation or autonomy in decision-making. |

**Table S4**. Mean values of physico-chemical parameters and chemical concentrations (including standard deviations for sites with *n*>1, where samples below detection limit are treated as zero) and Chemical Water Quality Hazard Rating (CWQHR) for samples sites in 2013 - 2014. Note: only one sample from many sites were analysed for Ba and Mo. EC; electrical conductivity, SAR; sodium adsorption ratio, TDS; total dissolved solids, irr.ch; irrigation channel.

| Sample site code (type)  (number of samples, *n*) | | | | *sa* (irr.ch)  ( *n* = 4) | *sb* (river)  (*n* = 3) | *sc* (river)  (*n* = 4) | *sd* (slope)  (*n* = 4) | *se* (irr.ch)  ( *n* = 4) | *sf* (river)  (*n* = 4) | *ga* (well)  (*n* = 2) | *gb* (well)  (*n* = 4) | *gc* (well)  (*n* = 1) |
| --- | --- | --- | --- | --- | --- | --- | --- | --- | --- | --- | --- | --- |
| Parameter  (mg/L unless  specified) | Detection  Limit  (mg/L) | Bolivian  'A'  criteria | CWQHR | 3 | 3 | 3 | 5 | 7 | 5 | 8 | 4 | 6 |
| pH | N/A | 6.0 – 8.5 | | 7.2 ± 0.4 | 7.3 ± 1.4 | 8.4 ± 0.3 | 7.6 ± 0.1 | 8.1 ± 0.3 | 8.7 ± 0.3 | 3.9 ± 0.3 | 6.9 ± 0.3 | 6.82 |
| EC (dS/m) | N/A | <1.5 ❶ | | 0.2 ± 0.0 | 0.3 ± 0.1 | 0.3 ± 0.0 | 1.4 ± 0.2 | 2.6 ± 0.2 | 1.7 ± 0.8 | 0.9 ± 0.5 | 0.8 ± 0.1 | 1.2 |
| SAR❷ | N/A |  | | 0.9 ± 0.6 | 1.0 ± 0.7 | 1.0 ± 0.6 | 7.6 ± 0.4 | 17.7 ± 7.5 | 6.8 ± 0.5 | 2.8 ± 0.5 | 1.4 ± 0.6 | 1.7 |
| TDS | N/A | 1000.0 | | 100 ± 20 | 170 ± 50 | 160 ± 22 | 720 ± 91 | 1300 ± 10.0 | 840 ± 400 | 440 ± 260 | 390 ± 65 | 600 |
| Alkalinity | N/A |  | | 52 ± 6.3 | 79 ± 5.2 | 95 ± 12 | 340 ± 40 | 210 ± 24 | 142 ± 15 | --- | 160 ± 25 | 140 |
| Cl | 0.097 | 250.0 | | 10 ± 1.3 | 12 ± 3.0 | 12 ± 0.4 | 210 ± 11 | 580 ± 62 | 370 ± 170 | 29 ± 8.1 | 38 ± 5.2 | 77 |
| F * | 0.083 | 0.6 – 1.7 | | 2.2 ± 0.9 | 2.1 ± 1.0 | 1.7 ± 1.0 | 2.8 ± 1.6 | 2.8 ± 1.2 | 2.8 ± 1.4 | 3.5 ± 0.01 | 3.2 ± 0.4 | --- |
| SO_4_ | 1.9 | 300.0 | | 44 ± 20 | 34 ± 8.0 | 47 ± 6.9 | 72 ± 3.4 | 42 ± 4.9 | 39 ± 2.1 | 330 ± 270 | 190 ± 33 | 370 |
| Al | 0.0312 | 0.2 | | 0.03 ± 0.04 | 0.02 ± 0.03 | < 0.031 | < 0.031 | 0.01 ± 0.02 | 0.01 ± 0.02 | 15 ± 16 | 0.01 ± 0.02 | 0.06 |
| As * | 0.0248 | 0.05 | | 0.007 ± 0.015 | < 0.025 | < 0.025 | 0.007 ± 0.013 | < 0.0248 | 0.009 ± 0.017 | 0.039 ± 0.009 | < 0.025 | 0.080 |
| B * | 0.0266 | 1.0 | | 0.32 ± 0.06 | 0.33 ± 0.03 | 0.42 ± 0.10 | 3.00 ± 0.06 | 3.0 ± 0.25 | 1.6 ± 0.19 | 0.97 ± 0.68 | 0.78 ± 0.02 | 1.19 |
| Ba * | 0.0003 | 0.7 ❸ | | 0.02 ± 0.005 | 0.02 | 0.02 ± 0.001 | 0.05 ± 0.005 | 0.26 ± 0.017 | 0.10 | --- | 0.04 ± 0.005 | 0.08 |
| Ca | 0.0054 | 200.0 | | 18 ± 5.3 | 24 ± 1.8 | 29 ± 4.3 | 32 ± 1.3 | 48 ± 2.1 | 47 ± 17.6 | 55 ± 48.7 | 82 ± 4.3 | 120 |
| Cd * | 0.0011 | 0.005 | | 0.001 ± 0.001 | 0.002 ± 0.002 | 0.001 ± 0.001 | 0.001 ± 0.001 | < 0.0011 | 0.001 ± 0.001 | 0.060 ± 0.04 | 0.001 ± 0.001 | 0.006 |
| Co | 0.0035 | 0.1 | | 0.001 ± 0.002 | < 0.0035 | 0.001 ± 0.002 | < 0.0035 | < 0.0035 | 0.001 ± 0.002 | 0.22 ± 0.19 | < 0.0035 | < 0.0035 |
| Cr (III) * | 0.0052 | 0.05 | | < 0.0052 | < 0.0052 | < 0.0052 | < 0.0052 | < 0.0052 | < 0.0052 | 0.004 ± 0.006 | < 0.0052 | < 0.0052 |
| Cu * | 0.0044 | 0.05 | | < 0.0044 | < 0.0044 | < 0.0044 | < 0.0044 | < 0.0044 | < 0.0044 | 1.1 ± 1.1 | < 0.0044 | < 0.0044 |
| Fe | 0.0019 | 0.3 | | 0.020 ± 0.026 | 0.003 ± 0.005 | 0.036 ± 0.009 | 0.13 ± 0.15 | 0.034 ± 0.021 | 0.012 ± 0.002 | 9.9 ± 5.9 | 0.88 ± 0.99 | 1.9 |
| K | 0.0298 | --- | | 1.5 ± 0.2 | 3.3 ± 2.4 | 2.7 ± 0.4 | 11 ± 0.8 | 45 ± 2.6 | 24 ± 8.1 | 20 ± 3.2 | 10 ± 1.3 | 18. |
| Li | 0.0029 | 2.5 ❹ | | < 0.0029 | < 0.0029 | < 0.0029 | 1.7 ± 0.10 | 3.4 ± 0.31 | 1.6 ± 0.50 | 2.0 ± 2.2 | 0.10 ± 0.02 | 0.45 |
| Mg | 0.0010 | 100.0 | | 6.7 ± 1.9 | 11 ± 1.1 | 12 ± 1.8 | 19 ± 1.3 | 11 ± 0.7 | 12 ± 5.7 | 14 ± 12 | 18 ± 1.4 | 35 |
| Mn | 0.0004 | 0.5 | | 0.003 ± 0.001 | 0.001 ± 0.001 | 0.048 ± 0.021 | 0.028 ± 0.006 | 0.062 ± 0.020 | 0.010 ± 0.005 | 0.76 ± 0.19 | 0.45 ± 0.16 | 0.78 |
| Mo | 0.0083 | 0.02 ❸ | | < 0.0083 | < 0.0083 | < 0.0083 | < 0.0083 | < 0.0083 | < 0.0083 | --- | <0.0083 | < 0.0083 |
| Na | 0.0047 | 200.0 | | 16 ± 5.1 | 23 ± 17 | 26 ± 15 | 220 ± 8.7 | 520 ± 225.2 | 200 ± 34 | 87 ± 55 | 54 ± 26 | 84 |
| Ni * | 0.0074 | 0.05 | | < 0.0074 | < 0.0074 | < 0.0074 | < 0.0074 | < 0.0074 | < 0.0074 | 0.25 ± 0.24 | < 0.0074 | < 0.0074 |
| Pb * | 0.0251 | 0.05 | | < 0.025 | < 0.025 | < 0.0251 | < 0.0251 | < 0.0251 | < 0.0251 | < 0.0251 | < 0.0251 | < 0.0251 |
| Sb * | 0.0192 | 0.01 | | < 0.019 | 0.010 ± 0.017 | < 0.0192 | 0.027 ± 0.019 | 0.015 ± 0.019 | < 0.0192 | 0.019 ± 0.027 | < 0.0192 | < 0.0192 |
| Si | 0.0263 | --- | | 8.3 ± 1.1 | 9.5 ± 0.8 | 9.2 ± 5.4 | 8.8 ± 0.4 | 13.5 ± 1.7 | 10.0 ± 1.8 | 14.6 ± 12.8 | 17.3 ± 0.9 | 18.1 |
| Sn | 0.0353 | 0.025 ❺ | | 0.011 ± 0.022 | 0.046 ± 0.047 | 0.033 ± 0.023 | 0.047 ± 0.038 | 0.045 ± 0.038 | < 0.0353 | 0.022 ± 0.031 | 0.063 ± 0.023 | 0.065 |
| Zn | 0.0026 | 0.2 | | 0.001 ± 0.003 | < 0.0026 | 0.003 ± 0.007 | 0.008 ± 0.015 | 0.001 ± 0.001 | <0.0026 | 2.09 ± 0.80 | 0.022 ± 0.028 | 0.009 |

❶ With respect to general recommendations for drinking water electrical conductivity; EC <0.9 dS/m, not recommended as palatable for human consumption >1.5 dS/m.

❷ Refer to FAO UN (1985) for information regarding soil infiltration and Sodium Adsorption Ratios; SAR = [Na meq/l]/({[Ca meq/l]+[Mg meq/l])/2})^1/2^

❸ With respect to WHO (2011) guidelines (see Table S5) as Bolivian class ‘A’ criteria is not known to exist.

❹ With respect to FAO recommendations for non-restricted use of irrigation water (Table S5).

| Sample site code (type)  (number of samples, *n*) | | | | *gd* (well)  (*n* = 4) | *ge* (well)  (*n* = 4) | *gf* (well)  (*n* = 2) | *gh* (well)  (*n* = 2) | *gi* (well)  (*n* = 4) | *gj* (well)  (*n* = 4) | *gg* (well)  (*n* = 4) | *tb* (well)  (*n* = 4) | *ta* (well)  (*n* = 4) |
| --- | --- | --- | --- | --- | --- | --- | --- | --- | --- | --- | --- | --- |
| Parameter  (mg/L unless  specified) | Detection  Limit  (mg/L) | Bolivian  'A'  criteria | CWQHR | 7 | 7 | 7 | 7 | 7 | 7 | 9 | 9 | 9 |
| pH | N/A | 6.0 – 8.5 | | 7.8 ± 0.4 | 7.1 ± 0.2 | 7.6 ± 0.6 | 7.1 ± 0.05 | 7.3 ± 0.4 | 7.7 ± 0.2 | 8.2 ± 0.2 | 6.7 ± 0.1 | 6.7 ± 0.1 |
| EC (dS/m) | N/A | <1.5 ❶ | | 3.4 ± 0.4 | 4.3 ± 0.5 | 3.2 ± 0.6 | 2.8 ± 0.5 | 5.0 ± 0.9 | 2.3 ± 0.1 | 16.3 ± 1.4 | 11.0 ± 1.7 | 6.5 ± 0.9 |
| SAR❷ | N/A |  | | 9.9 ± 5.8 | 14 ± 8.9 | 15 ± 8.6 | 5.5 ± 0.9 | 14 ± 7.6 | 6.0 ± 0.9 | 47.6 ± 25.7 | 50 ± 10 | 38 ± 15 |
| TDS | N/A | 1000.0 | | 1700 ± 180 | 2100 ± 230 | 1600 ± 300 | 1400 ± 240 | 2500 ± 450 | 1200 ± 68 | 8100 ± 690 | 5300 ± 710 | 3300.0 ± 440 |
| Alkalinity | N/A |  | | 330 ± 45 | 390 ± 36 | 310 ± 39 | 100 ± 16 | 260 ± 40 | 150 ± 33 | 510 ± 90 | 390 ± 18 | 410 ± 34 |
| Cl | 0.097 | 250.0 | | 710 ± 80 | 1000 ± 81 | 620 ± 44 | 270 ± 39 | 1000 ± 150 | 270 ± 26 | 3900 ± 1400 | 2800 ± 130 | 1600 ± 140 |
| F * | 0.083 | 0.6 – 1.7 | | 2.1 ± 1.2 | 2.5 ± 0.8 | 3.5 ± 0.7 | 3.4 ± 0.3 | 3.2 ± 1.0 | 3.3 ± 1.1 | 3.6 ± 0.4 | 4.1 ± 1.3 | 3.3 ± 2.1 |
| SO_4_ | 1.9 | 300.0 | | 130 ± 41 | 100 ± 14 | 77 ± 8.3 | 840 ± 55 | 540 ± 77 | 610 ± 92 | 74 ± 58 | 61 ± 5.2 | 36 ± 11 |
| Al | 0.0312 | 0.2 | | 0.01 ± 0.02 | 0.02 ± 0.02 | < 0.031 | < 0.031 | 0.03 ± 0.03 | < 0.031 | 0.01 ± 0.02 | 0.03 ± 0.03 | 0.01 ± 0.02 |
| As * | 0.0248 | 0.05 | | < 0.025 | 0.018 ± 0.022 | < 0.025 | 0.10 ± 0.03 | 0.059 ± 0.005 | 0.11 ± 0.02 | 0.099 ± 0.077 | < 0.025 | < 0.025 |
| B * | 0.0266 | 1.0 | | 3.0 ± 0.17 | 4.6 ± 0.83 | 3.3 ± 0.10 | 2.4 ± 0.21 | 4.1 ± 0.93 | 2.0 ± 0.18 | 14 ± 0.84 | 8.5 ± 0.30 | 6.7 ± 0.19 |
| Ba * | 0.0003 | 0.7 ❸ | | 0.16 | 0.28 ± 0.028 | --- | --- | 0.04 | 0.04 ± 0.013 | 0.31 | High | High |
| Ca | 0.0054 | 200.0 | | 180 ± 34.1 | 180 ± 28 | 110 ± 11 | 190 ± 12 | 250 ± 22.0 | 140 ± 17.3 | 160 ± 25 | 100 ± 3.1 | 82 ± 3.2 |
| Cd * | 0.0011 | 0.005 | | 0.001 ± 0.001 | < 0.0011 | < 0.0011 | < 0.0011 | 0.002 ± 0.003 | 0.002 ± 0.002 | < 0.0011 | 0.001 ± 0.002 | 0.001 ± 0.001 |
| Co | 0.0035 | 0.1 | | < 0.0035 | < 0.0035 | < 0.0035 | < 0.0035 | < 0.0035 | < 0.0035 | 0.001 ± 0.002 | < 0.0035 | < 0.0035 |
| Cr (III) * | 0.0052 | 0.05 | | < 0.0052 | < 0.0052 | < 0.0052 | < 0.0052 | < 0.0052 | < 0.0052 | < 0.0052 | < 0.0052 | < 0.0052 |
| Cu * | 0.0044 | 0.05 | | < 0.0044 | < 0.0044 | < 0.0044 | < 0.0044 | < 0.0044 | 0.002 ± 0.003 | < 0.0044 | 0.007 ± 0.009 | < 0.0044 |
| Fe | 0.0019 | 0.3 | | 0.005 ± 0.001 | 0.016 ± 0.008 | 0.008 ± 0.002 | 0.009 ± 0.0004 | 0.010 ± 0.004 | 0.042 ± 0.038 | 0.080 ± 0.052 | 0.80 ± 0.29 | 0.45 ± 0.10 |
| K | 0.0298 | --- | | 56 ± 5.9 | 66 ± 6.9 | 52 ± 3.0 | 36 ± 0.4 | 62 ± 16 | 31 ± 1.2 | 270 ± 30 | 150 ± 4.7 | 120 ± 1.9 |
| Li | 0.0029 | 2.5 ❹ | | 3.5 ± 0.25 | 4.6 ± 0.57 | 3.0 ± 0.37 | 2.1 ± 0.01 | 2.9 ± 0.85 | 1.8 ± 0.15 | 17 ± 2.6 | 10 ± 0.83 | 7.1 ± 4.8 |
| Mg | 0.0010 | 100.0 | | 38 ± 5.1 | 45 ± 7.5 | 30 ± 5.2 | 40 ± 0.6 | 47 ± 10.4 | 25 ± 1.3 | 29 ± 3.2 | 13 ± 0.9 | 12 ± 0.9 |
| Mn | 0.0004 | 0.5 | | 0.001 ± 0.001 | 0.054 ± 0.073 | 0.002 ± 0.0001 | 0.025 ± 0.001 | 1.3 ± 0.64 | 4.1 ± 2.2 | 0.44 ± 0.32 | 0.37 ± 0.016 | 0.18 ± 0.009 |
| Mo | 0.0083 | 0.02 ❸ | | < 0.0083 | < 0.0083 | --- | --- | < 0.0083 | 0.003 ± 0.005 | < 0.0083 | < 0.0083 | < 0.0083 |
| Na | 0.0047 | 200.0 | | 550 ± 300 | 800 ± 460 | 710 ± 440 | 320 ± 58 | 920 ± 470 | 290 ± 50 | 2500 ± 1300 | 2000 ± 440 | 1400 ± 550 |
| Ni * | 0.0074 | 0.05 | | < 0.0074 | < 0.0074 | < 0.0074 | < 0.0074 | < 0.0074 | < 0.0074 | < 0.0074 | < 0.0074 | < 0.0074 |
| Pb * | 0.0251 | 0.05 | | 0.0078 ± 0.016 | < 0.0251 | 0.014 ± 0.019 | < 0.025 | < 0.025 | < 0.025 | < 0.0251 | < 0.0251 | 0.017 ± 0.020 |
| Sb * | 0.0192 | 0.01 | | < 0.019 | < 0.019 | < 0.019 | 0.020 ± 0.029 | 0.045 ± 0.091 | 0.040 ± 0.081 | 0.022 ± 0.027 | 0.015 ± 0.018 | 0.059 ± 0.092 |
| Si | 0.0263 | --- | | 17 ± 3.7 | 17 ± 0.7 | 16 ± 1.4 | 13 ± 1.3 | 12 ± 2.1 | 18 ± 3.7 | 18 ± 2.4 | 34 ± 1.7 | 22 ± 6.0 |
| Sn | 0.0353 | 0.025 ❺ | | 0.067 ± 0.031 | 0.059 ± 0.012 | 0.059 ± 0.083 | 0.069 ± 0.015 | 0.072 ± 0.058 | 0.058 ± 0.016 | 0.044 ± 0.052 | 0.050 ± 0.039 | 0.036 ± 0.026 |
| Zn | 0.0026 | 0.2 | | < 0.0026 | < 0.0026 | 0.013 ± 0.015 | 0.020 ± 0.005 | 0.009 ± 0.006 | 0.027 ± 0.015 | 0.019 ± 0.019 | 0.008 ± 0.003 | 0.008 ± 0.006 |

| Sample site code (type)  (number of samples, *n*) | | | | *ma* (slope)  (*n* = 4) | *mb* (river)  (*n* = 4) | *mc* (river)  (*n* = 4) | *md* (river)  (*n* = 4) | *me* (river)  (*n* = 4) | *mf* (river)  (*n* = 4) | *mg* (river)  (*n* = 1) | *mh* (river)  (*n* = 4) | *mi* (river)  (*n* = 4) |
| --- | --- | --- | --- | --- | --- | --- | --- | --- | --- | --- | --- | --- |
| Parameter  (mg/L unless  specified) | Detection  Limit  (mg/L) | Bolivian  'A'  criteria | CWQHR | 6 | 8 | 8 | 8 | 8 | 8 | 6 | 8 | 8 |
| pH | N/A | 6.0 – 8.5 | | 7.6 ± 0.9 | 4.5 ± 1.3 | 4.4 ± 1.2 | 3.5 ± 0.5 | 3.3 ± 0.1 | 3.8 ± 0.4 | 7.31 | 4.3 ± 0.6 | 4.6 ± 0.8 |
| EC (dS/m) | N/A | <1.5 ❶ | | 0.4 ± 0.1 | 2.9 ± 0.4 | 3.0 ± 0.2 | 3.2 ± 0.1 | 2.7 ± 0.4 | 2.2 ± 0.4 | 1.5 | 2.2 ± 0.6 | 2.2 ± 0.6 |
| SAR❷ | N/A |  | | 1.3 ± 0.6 | 0.9 ± 0.1 | 0.9 ± 0.2 | 0.9 ± 0.2 | 1.0 ± 0.3 | 1.0 ± 0.3 | 1.2 | 1.3 ± 0.2 | 1.8 ± 0.2 |
| TDS | N/A | 1000.0 | | 210 ± 25 | 150 ± 220 | 1500 ± 120 | 1600 ± 40 | 1400 ± 220 | 1100 ± 220 | 770 | 1100 ± 320 | 1100.5 ± 290 |
| Alkalinity | N/A |  | | 48 ± 7.7 | 11 | 5.8 | --- | --- | --- | 45.0 | --- | --- |
| Cl | 0.097 | 250.0 | | 13 ± 2.8 | 160 ± 87 | 170 ± 80 | 170 ± 76 | 120 ± 26 | 100 ± 18 | 89.0 | 130 ± 20 | 170 ± 48 |
| F * | 0.083 | 0.6 – 1.7 | | 2.4 ± 1.0 | 3.0 ± 0.9 | 3.2 ± 0.9 | 3.2 ± 1.1 | 2.0 ± 1.2 | 2.8 ± 1.2 | 1.4 | 2.9 ± 1.3 | 2.4 ± 1.9 |
| SO_4_ | 1.9 | 300.0 | | 130 ± 40 | 1400 ± 410 | 1500 ± 320 | 1400 ± 390 | 1100 ± 460 | 980 ± 190 | 650 | 910 ± 200 | 850 ± 190 |
| Al | 0.0312 | 0.2 | | 0.05 ± 0.10 | 13 ± 10 | 14 ± 12 | 21 ± 9.3 | 28 ± 5.3 | 19 ± 6.2 | 0.07 | 16 ± 7.6 | 9.7 ± 6.5 |
| As * | 0.0248 | 0.05 | | 0.013 ± 0.025 | 0.017 ± 0.034 | 0.042 ± 0.057 | 0.41 ± 0.41 | 0.007 ± 0.015 | < 0.025 | < 0.025 | < 0.025 | < 0.025 |
| B * | 0.0266 | 1.0 | | 0.69 ± 0.13 | 0.32 ± 0.06 | 0.37 ± 0.04 | 0.41 ± 0.04 | 0.44 ± 0.04 | 0.44 ± 0.06 | 0.91 | 0.62 ± 0.12 | 0.75 ± 0.17 |
| Ba * | 0.0003 | 0.7 ❸ | | 0.04 | 0.07 | 0.06 | 0.03 | 0.01 | 0.01 | 0.04 | 0.02 | 0.03 |
| Ca | 0.0054 | 200.0 | | 38 ± 11 | 560 ± 260 | 600 ± 230 | 560 ± 160 | 400 ± 75 | 330 ± 67 | 170 | 320 ± 74 | 290 ± 75.2 |
| Cd * | 0.0011 | 0.005 | | 0.004 ± 0.002 | 0.35 ± 0.14 | 0.34 ± 0.12 | 0.34 ± 0.089 | 0.35 ± 0.037 | 0.29 ± 0.031 | 0.006 | 0.26 ± 0.035 | 0.23 ± 0.035 |
| Co | 0.0035 | 0.1 | | < 0.0035 | 0.037 ± 0.021 | 0.042 ± 0.021 | 0.064 ± 0.015 | 0.12 ± 0.015 | 0.084 ± 0.024 | 0.004 | 0.076 ± 0.026 | 0.064 ± 0.031 |
| Cr (III) * | 0.0052 | 0.05 | | < 0.0052 | 0.003 ± 0.007 | 0.003 ± 0.007 | 0.003 ± 0.007 | < 0.0052 | < 0.0052 | < 0.0052 | < 0.0052 | < 0.0052 |
| Cu * | 0.0044 | 0.05 | | < 0.0044 | 0.12 ± 0.12 | 0.13 ± 0.13 | 0.99 ± 0.55 | 0.59 ± 0.045 | 0.48 ± 0.047 | < 0.0044 | 0.42 ± 0.086 | 0.39 ± 0.12 |
| Fe | 0.0019 | 0.3 | | 0.034 ± 0.064 | 22 ± 43 | 24 ± 45 | 56 ± 29 | 2.0 ± 0.5 | 2.3 ± 1.8 | 0.05 | 1.8 ± 0.9 | 1.2 ± 0.8 |
| K | 0.0298 | --- | | 2.9 ± 0.3 | 12 ± 5.0 | 13 ± 4.7 | 11 ± 3.7 | 8.6 ± 1.0 | 8.2 ± 1.1 | 19 | 11 ± 0.3 | 12 ± 2.0 |
| Li | 0.0029 | 2.5 ❹ | | < 0.0029 | 0.17 ± 0.08 | 0.19 ± 0.08 | 0.23 ± 0.10 | 0.40 ± 0.07 | 0.34 ± 0.06 | 1.0 | 0.54 ± 0.07 | 0.61 ± 0.15 |
| Mg | 0.0010 | 100.0 | | 12 ± 3.2 | 18 ± 4.0 | 19 ± 2.6 | 21 ± 2.4 | 29 ± 1.3 | 27 ± 3.9 | 52 | 28 ± 3.7 | 27 ± 3.2 |
| Mn | 0.0004 | 0.5 | | 0.003 ± 0.004 | 2.9 ± 1.6 | 3.0 ± 1.4 | 3.9 ± 0.70 | 16 ± 2.1 | 10 ± 3.2 | 0.23 | 10 ± 3.7 | 7.5 ± 2.3 |
| Mo | 0.0083 | 0.02 ❸ | | < 0.0083 | 0.009 | < 0.0083 | < 0.0083 | < 0.0083 | < 0.0083 | < 0.0083 | < 0.0083 | < 0.0083 |
| Na | 0.0047 | 200.0 | | 33 ± 10 | 74 ± 12 | 82 ± 8.2 | 81 ± 7.9 | 74 ± 28 | 68 ± 26 | 68 | 91 | 120 ± 12.0 |
| Ni * | 0.0074 | 0.05 | | < 0.0074 | 0.074 ± 0.041 | 0.073 ± 0.037 | 0.12 ± 0.018 | 0.18 ± 0.013 | 0.16 ± 0.041 | 0.20 | 0.14 ± 0.029 | 0.13 ± 0.027 |
| Pb * | 0.0251 | 0.05 | | < 0.025 | 0.024 ± 0.048 | 0.021 ± 0.041 | 0.023 ± 0.031 | < 0.025 | < 0.025 | < 0.025 | < 0.0251 | < 0.025 |
| Sb * | 0.0192 | 0.01 | | 0.008 ± 0.015 | 0.018 ± 0.025 | 0.009 ± 0.017 | 0.020 ± 0.026 | < 0.019 | < 0.019 | < 0.019 | 0.049 ± 0.058 | 0.011 ± 0.012 |
| Si | 0.0263 | --- | | 15 ± 5.9 | 9.5 ± 4.3 | 9.4 ± 3.7 | 12 ± 3.5 | 27 ± 2.9 | 16 ± 11 | 30 | 23.0 ± 1.3 | 20 ± 4.1 |
| Sn | 0.0353 | 0.025 ❺ | | 0.049 ± 0.037 | 0.039 ± 0.029 | 0.041 ± 0.029 | 0.026 ± 0.053 | 0.024 ± 0.029 | 0.015 ± 0.029 | 0.075 | 0.037 ± 0.026 | 0.060 ± 0.025 |
| Zn | 0.0026 | 0.2 | | 0.60 ± 0.50 | 66 ± 36 | 70 ± 32 | 150 ± 160 | 190 ± 170 | 78 ± 16 | 39 | 75 ± 24 | 58 ± 12 |

**Table S5**. Guideline limit concentrations for water constituents and quality indicators for: Bolivian class A, B, C, and D criteria for receiving waters, WHO (2011) guidelines for drinking water, and Food and Agriculture Organization (FAO UN, 1985) recommended limits for livestock and non-restricted water use in agriculture. All in mg/L except pH and EC (dS/m).

| Parameter (mg/L unless other stated) | Bolivian class A | Bolivian class B | Bolivian class C | Bolivian class D | WHO guideline | FAO recommendation for livestock | FAO recommendation for non-restricted irrigation use |
| --- | --- | --- | --- | --- | --- | --- | --- |
| pH | pH 6 – 8.5 | pH 6 – 9 | pH 6 – 9 | pH 6 – 9 | pH 6.5 – 8.5 | --- | pH 6.5 - 8.4 |
| Electrical conductivity (EC, dS/m)❶ | <1.5dS/m❶ | --- | --- | --- | (<0.9dS/m) <1.5dS/m❶ | <5-8dS/m (<5 poultry) | <0.7 dS/m (>3 dS/m severe restriction) |
| TDS | 1000 | 1000 | 1500 | 1500 | (600) 1000 | 5100 (3200) | <450 good (>2000 severe restriction) |
| SAR (unit less) ❷ | --- | --- | --- | --- | --- | --- | 0-3, EC>0.7dS/m; 3-6, EC >1.2 dS/m❷ |
| Cl, chloride | 250 | 300 | 400 | 500 | 250 | --- | 140 (>350 severe) (as ion toxicity) |
| F, fluoride | 0.6 – 1.7 | --- | --- | --- | 1.5^ | 2.0 | 1.0 |
| SO_4_, sulphate | 300 | 400 | 400 | 400 | 500 | --- | 500 |
| Al, aluminium | 0.2 | 0.5 | 1.0 | 1.0 | 0.2 | 5.0 | 5.0 |
| As, arsenic | 0.05 | 0.05 | 0.05 | 0.1 | 0.01^^ | 0.2 | 0.1 |
| B, boron | 1.0 | 1.0 | 1.0 | 1.0 | 2.4^ | 5.0 | 0.7 (>3 severe) |
| Ba, barium | --- | --- | --- | --- | 0.7^ | --- | --- |
| Ca, calcium | 200 | 300 | 300 | 400 | --- | --- | --- |
| Cd, cadmium | 0.005 | 0.005 | 0.005 | 0.005 | 0.003^^^ | 0.05 | 0.01 |
| Co, cobalt | 0.1 | 0.2 | 0.2 | 0.2 | --- | 1.0 | 0.05 |
| Cr, chromium (III) | 0.05 | 0.6 | 0.6 | 1.1 | 0.05^^ | 1.0 | 0.1 |
| Cu, copper | 0.05 | 1.0 | 1.0 | 1.0 | 2.0^ | 2.0 | 0.2 |
| Fe, iron | 0.3 | 0.3 | 1.0 | 1.0 | --- | --- | 5.0 |
| K, potassium | --- | --- | --- | --- | --- | --- | --- |
| Li, lithium | --- | --- | --- | --- | --- | --- | 2.5 |
| Mg, magnesium | 100 | 100 | 150 | 150 | --- | 250 (cattle 400) | --- |
| Mn, manganese | 0.5 | 1.0 | 1.0 | 1.0 | 0.4 | --- | 0.2 |
| Mo, molybdenum | --- | --- | --- | --- | 0.02 | --- | 0.01 |
| Na, sodium | 200 | 200 | 200 | 200 | 200 | --- | 69 (>206 severe) (as ion toxicity) |
| Ni, nickel | 0.05 | 0.05 | 0.5 | 0.5 | 0.07^ | --- | 0.2 |
| Pb, lead | 0.05 | 0.05 | 0.05 | 0.1 | 0.01^^ | 0.1 | 5.0 |
| Sb, antimony | 0.01 | 0.01 | 0.01 | 0.01 | 0.02^ | --- | --- |
| Si, silica | --- | --- | --- | --- | --- | --- | --- |
| Sn, tin ❸ | 0.025❸ | --- | --- | --- | --- | --- | --- |
| Zn, zinc | 0.2 | 0.2 | 5.0 | 5.0 | 3.0 | 24.0 | 2.0 |

❶ Generally, recommended drinking water EC <0.9 dS/m and at maximum 1.5 dS/m (i.e., <600 ppm TDS and maximum 1000 ppm TDS as recommended by the WHO (2011) for taste and palatability). EC >2.5 dS/m is not recommended for consumption and that with >10 dS/m is considered not for consumption (livestock included).

❷ Sodium Adsorption Ratio (SAR); defines sodicity in terms of the relative concentration of sodium (Na) compared to the sum of calcium (Ca) and magnesium (Mg) ions. The SAR assesses the potential for infiltration problems due to a sodium imbalance in irrigation water. Recommendations by the FAO UN (1985) for non-restricted use as irrigation water are given to avoid infiltration problems depending on associated EC and local soil type/condition. SAR = [Na meq/l] / ({[Ca meq/l] + [Mg meq/l])/2})^1/2^

^ Element of health significance in WHO guidelines (2011). Guidelines for other elements not included generally refer to acceptability for taste, odour, scaling etc. (NB. Mn guideline is based on intake assessment, and Al guideline is based on use in water treatment flocculation despite possible health concerns).

^^ Provisional WHO health-based guideline value set higher (i.e., as achievable) than initially calculated value which was below i) the achievable quantification level, and ii) the level achievable through practical treatment etc. (WHO, 2011).

^^^ Provisional health-based WHO guideline value due to scientific uncertainty (WHO, 2011).

❸ UK Environment Agency non-statutory recommended limit for protection of aquatic life.

| **Table S6**. Qualitative description of Chemical^^[[1]](#footnote-1)^^ Water Quality Hazard Rating (CWQHR) and categorisation of assessed sites. | | |
| --- | --- | --- |
| CWQHR | Qualitative description | Assessed sites: |
| **1** | Chemically and biologically of good quality for consumption and other uses. Undergone any necessary treatment for potable water. | --- |
| **2** | Chemically good for consumption and other uses (excluding assessment of industrial, agricultural, and petroleum chemicals), pending microbial assessment. | --- |
| **3** | Chemically good for consumption (electrical conductivity, EC, <0.5 dS/m, sodium adsorption ratio, SAR, <3); meeting Bolivian class 'A' criteria with the exception of a maximum of two elements that have health-based World Health Organization (WHO) guideline (referred to here as element of health significance) that sometimes exceed 'A' criteria (or WHO if no 'A' criteria exists) but not to excessive concentrations (i.e., Hazard Quotient, HQ; sample element concentration/Bolivian 'A' criteria = <3), and occasional/seasonal appearance factors (e.g., some algae or slightly turbid at times). Good for irrigation but infiltration may be problematic due to combination of low EC and SAR. Microbial assessment required. Treatment to reduce elements exceeding health-based guidelines recommended in addition to basic treatment for potable water (e.g., filtration, disinfection). | *sa, sb, sc* |
| **4** | Chemically acceptable with EC <0.9 dS/m but with a maximum of three elements that exceed Bolivian 'A' criteria (or WHO guideline if no 'A' criteria exists) but not excessively, especially for any element of health significance (i.e., Hazard Quotient, HQ <3). Possibly concern over the presence of algae, suspended particulate/organic material such as animal droppings, and/or stagnation, which may suggest poor microbial quality. Water generally good for livestock, and not too bad for human consumption with caution due to element of health significance and pending microbial status. Suitable for irrigation but infiltration may be problematic due to combination of low EC and SAR. Microbial assessment required and actions such as cleaning of tanks/pools, pumping and covering of wells. Treatment to reduce element of health significance such as fluoride recommended in addition to basic treatment for potable water (e.g., filtration, disinfection). | *gb* |
| **5** | Reduced quality in comparison to CWQHR 4 due to higher electrical conductivity (1 - 2 dS/m) in addition to caution over human consumption due to (naturally sourced) elements of health significance exceeding 'A' criteria (or WHO guideline if no 'A' criteria exists), and concern over the presence of algae, suspended particulate/organic material such as animal droppings, and/or stagnation, which may suggest poor microbial quality. Not recommended for human consumption due to EC and any element of health significance exceedances. Acceptable for most livestock and not too bad for irrigation, but infiltration may be problematic when EC <1.2 dS/m when SAR 3 - 6. | *sd, sf* |
| **6** | Quality issues due to general exceedance of many Bolivian 'A' criteria and often 'B-D', thus numerous elements (mining and/or naturally sourced) Hazard Quotient (HQ) >1 and often >5. May be affected by mine water infiltration or migration, and is not therefore recommended for human consumption for this reason and due to concern over microbial status. May be acceptable for livestock with caution (depending on elements with exceedance possibly only for higher tolerant livestock). Some waters may be acceptable for irrigation depending on elements HQ >1, but infiltration maybe problematic if EC <0.7 dS/m when SAR 0 - 3, EC <1.2 dS/m when SAR 3 - 6, EC <1.9 dS/m when SAR 6 - 12. | *gc, ma, mg* |
| **7** | Water with naturally high salts, of very poor quality for human (and lower tolerant livestock) consumption and not recommended for irrigation use due to high EC (>1.5 dS/m) and TDS (>1000 mg/L) in addition to >2 elements of health significance exceeding 'A' criteria (or WHO guideline if no 'A' criteria) and FAO recommendations. With numerous elements HQ >1, and possibly concerns over biological quality. Not recommended for consumption without significant treatment (e.g., desalination, reduction of metals). | *se, gd, ge, gf, gh, gi. gj* |
| **8** | Mine affected water. Unsuitable for any use due to exceedance of 'A-D' criteria for sulphate and >4 metals that have high to very high concentrations (at least two with HQ>10), >2 metals being elements of health significance, and high EC (>1.5 dS/m). Not suitable for any use without significant treatment (e.g., desalination, removal of metals). | *ga, mb, mc, md, me,* *mf, mh, mi* |
| **9** | Thermal waters that can >60 ⁰C. Unsuitable for human or animal consumption or irrigation/agriculture because of naturally very high concentrations of salts (EC >8 dS/m) in addition to high concentrations of Li and elements of health significance; boron, fluoride and antimony. Recreational use as bathing waters. | *ta, tb, gg* |
| **10** | High level mine affected water. Complete restriction on use for humans, animal or irrigation/agriculture because of exceedance of 'A-D' criteria for sulphate and >2 metals that have extremely high concentrations (HQ>100), >3 other metals HQ >5 (>4 being elements of health significance), and very high EC (>8). Many elements HQ>100. Waters that require significant remediation for major reduction of many metals with subsequent desalination. | --- |

1. Chemical refers to naturally present elements and those sourced from mining activity and excludes chemicals sourced from agriculture (herbicides, pesticides etc.), petroleum- and industrially-derived chemicals (solvents, plastic related etc.), and water treatment chemicals (chlorination by products etc.). [↑](#footnote-ref-1)
